# Contribution of sarcomere gene mutations to left atrial function in patients with hypertrophic cardiomyopathy

**DOI:** 10.1186/s12947-020-00233-y

**Published:** 2021-01-06

**Authors:** Hyemoon Chung, Yoonjung Kim, Chul Hwan Park, In-Soo Kim, Jong-Youn Kim, Pil-Ki Min, Young Won Yoon, Tae Hoon Kim, Byoung Kwon Lee, Bum-Kee Hong, Se-Joong Rim, Hyuck Moon Kwon, Kyung-A Lee, Eui-Young Choi

**Affiliations:** 1 Department of Internal Medicine, Division of Cardiology, Kyung Hee University School of Medicine, Seoul, South Korea; 2 Department of Laboratory Medicine, Gangnam Severance Hospital, Yonsei University College of Medicine, 211 Eonju-Ro, Gangnam-Gu, Seoul, 06273 Republic of Korea; 3 Department of Radiology, Gangnam Severance Hospital, Yonsei University College of Medicine, Seoul, South Korea; 4 Division of Cardiology, Gangnam Severance Hospital, Yonsei University College of Medicine, 211 Eonju-Ro, Gangnam-Gu, Seoul, 06273 Republic of Korea

**Keywords:** Hypertrophic cardiomyopathy, Genetic mutation, Left atrial function

## Abstract

**Background:**

Left atrial (LA) enlargement and dysfunction are related to clinical course in patients with hypertrophic cardiomyopathy (HCM). We aimed to investigate genetic contribution to LA structural and functional remodeling.

**Methods:**

Two hundred twelve patients were consecutively enrolled, and echocardiography and extensive genetic analysis were performed. Cardiac magnetic resonance (CMR) was performed in 135 patients. Echocardiography was also performed in controls (*n* = 30).

**Results:**

Patients with HCM had lower late-diastolic mitral annular velocity (a’) and higher LA volume index (LAVI) than controls. Patients with pathogenic or likely pathogenic sarcomere gene mutations (PSM, *n* = 67, 32%) had higher LAVI and lower CMR-derived LA total emptying fraction (37.0 ± 18.5 vs. 44.2 ± 12.4%, *p* = 0.025). In patients without AF (*n* = 187), the PSM had lower a’ (6.9 ± 2.0 vs. 7.8 ± 1.9 cm/s, *p* = 0.004) than others. The PSM had higher prevalence and amount of late gadolinium enhancement (LGE) in the left ventricle (LV). In multivariate analysis, PSM was significantly related to lower a’ independent of E/e’, LV mass index, and LAVI. However, the relation significantly attenuated after adjustment for the extent of LGE in the LV, suggesting common myopathy in the LV and LA. In addition, PSM was significantly related to lower LA total emptying fraction independent of age, E/e’, s’, LV ejection fraction, LV myocardial global longitudinal strain and %LGE mass.

**Conclusions:**

PSM was related to LA dysfunction independent of LV filling pressure and LAVI, suggesting its contribution to atrial myopathy in HCM.

**Supplementary Information:**

The online version contains supplementary material available at 10.1186/s12947-020-00233-y.

## Background

Left atrial (LA) enlargement and dysfunction are related to the development of atrial fibrillation (AF) and exercise intolerance in patients with hypertrophic cardiomyopathy (HCM) [1–3]. LA dysfunction is usually related to chronic structural remodeling owing to long-standing pressure and volume overload. In addition, LA structural remodeling such as enlargement or fibrosis is a marker of development of AF in HCM [4]. Recent studies also showed that LA function measured by strain imaging is an independent predictor of poor outcome in patients with HCM [1, 2]. However, it has been reported that in HCM, LA dysfunction can occur as a myopathic process itself, irrespective of loading conditions [4, 5]. In this regard, genetic contribution to LA structural and functional remodeling has not been well investigated. Tissue Doppler imaging-based late mitral annular velocity (a’) and LA phasic function have been validated as good LA functional parameters reflecting active contraction in several studies and are widely used [6]. Herein, we sought to evaluate the relationship between related gene mutations and LA function in patients with HCM. In addition, we aimed to study the direct effect on LA function as a process of LA myopathy, independent of LA loading conditions.

## Materials and methods

### Study population

A total of 432 patients treated at a single center were enrolled in an HCM Registry from 2006 to 2014. Among them, 220 patients were excluded owing to insufficient data, follow-up loss, or declining study enrollment. Finally, 212 patients underwent genetic testing. The patients had maximal left ventricular (LV) hypertrophy greater than 13 mm and a ratio of maximal thickness to posterior wall thickness greater than 1.3 without an underlying cause of hypertrophy, such as uncontrolled hypertension or aortic stenosis. Patterns of LV hypertrophy were classified as ApHCM and non-ApHCM (asymmetrical hypertrophy, diffuse hypertrophy, and focal segmental hypertrophy). All patients underwent screening for Fabry disease and were confirmed negative for the galactosidase alpha variant. For comparison, conventional echocardiography was performed in controls. The study protocol conformed to the ethical guidelines of the 1975 Declaration of Helsinki, and it was approved by institutional review board of Gangnam Severance Hospital (3–2015-0019). Written informed consent was obtained from all participants.

### Genetic testing and analysis

#### HCM gene panel (nDNA) and mtDNA design

HCM genes consisted of 8 validated sarcomere genes and 25 putative HCM genes [7]. A comprehensive HCM-specific panel, consisting of 82 nuclear DNAs (nDNAs: 33 sarcomere-associated genes, 5 phenocopy genes, and 44 nuclear genes linked to mitochondrial cardiomyopathy) and 37 mitochondrial DNAs (mtDNAs), was analyzed (Supplementary Table S1).

#### DNA preparation, library construction and sequencing of HCM gene panel and mtDNA

The details are described in Supplementary Method 1 to 3.

#### Identification of potential pathogenic mtDNA variants

Mitochondrial genome databases, including MITOMAP [8] and Human Mitochondrial Genome Database (mtDB) [9] and Phylotree [10] were referred to validate the detected variants. Novel and rare non-haplogroup-associated variants were further evaluated for their potential pathogenicity based on the variant’s location, changes in the amino acid sequence, and evolutionary conservation [11]. We have assessed potential pathogenicity using multiple software programs including Polyphen2, Fathmmw, Mutation Assessor, and PROVEAN. When the majority of computational evidence supported a deleterious effect, we have assigned novel and rare non-haplogroup-associated variants as damaging mtDNA variants [12]. Data analysis of mitochondrial genome are described in Supplementary Method 4.

#### Data analysis of the HCM gene panel

The Burrows–Wheeler aligner algorithm with default parameters was used to align reads to the human reference genome sequence GRCh37 [13]. SAMTools was used to convert the sequence alignment map file to the BAM format [14]. Duplicates were sorted and removed using the Picard tool (http://broadinstitute.github.io/picard/). The Genome Analysis Toolkit was used for indel realigning and base-quality score re-calibration [15]. Variants were further filtered with altered allele frequency > 30%, 50× coverage. For 33 HCM genes, annotated variants using ANNOVAR [16] and Variant Effect Predictor (http://asia.ensembl.org/info/docs/tools/vep/index.html) were classified as pathogenic and likely pathogenic based on refined American College of Medical Genetics and Genomics (ACMG) standards and guidelines for inherited cardiac conditions [12, 17–19]. For 44 mitochondria-related nuclear DNA genes (recessive conditions), annotated variants were classified as pathogenic and likely pathogenic based on ACMG guidelines [17]. And we adapted gnomAD AF cutoff 0.01% as the moderate level of evidence supporting pathogenicity (ACMG/AMP criterion PM2) based on maximum credible population AF (http://cardiodb.org/allelefrequencyapp/) [17, 18].

### Echocardiographic analysis

**Fig. 1:**
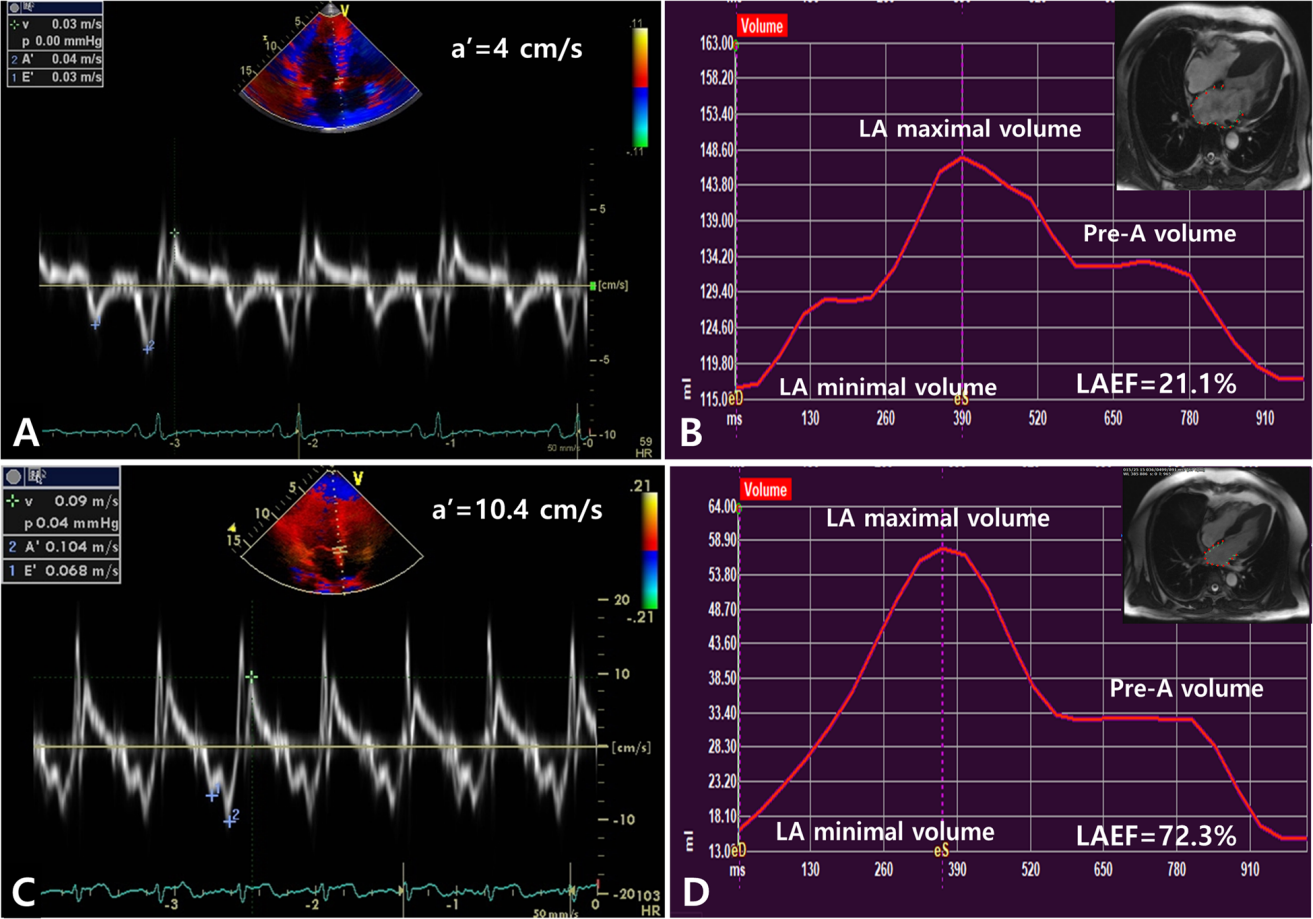
Tissue Doppler index (right panel) and phasic left atrial function measurement with cardiac magnetic resonance imaging (right panel) in a patient with pathogenic sarcomere gene mutation (*TNNI3* mutation, **a**, **b**) and without sarcomere mutations (**c**, **d**)

Comprehensive echo-Doppler evaluation was performed according to the current American Society of Echocardiography guidelines [20]. LV ejection fraction was measured by biplane Simpson’s method. LA volume was measured at the end-systole by the ellipsoidal method, and LA volume index was calculated as LA volume/body surface area (BSA). Peak early (E) and late (A) diastolic mitral inflow velocities were measured in apical four-chamber view. The filter was set to exclude high frequency signal, and the Nyquist limit was adjusted to a range of 15 to 20 cm/s. Gain and sample volume were minimized to allow for a clear tissue signal with minimal background noise. Systolic (s’), early (e’) and late (a’) diastolic velocities of the mitral annulus were measured from the apical 4-chamber view with a 2- to 5-mm sample volume placed at the septal corner of the mitral annulus. (Fig. 1) The ratio of E/e′ was calculated. LV wall thickness was measured in all cross-sectional planes. Continuous wave Doppler was used to measure the peak velocity across the LV outflow tract (LVOT), and the pressure gradient was calculated using the Bernoulli equation, as follows: 4 × (peak velocity across the LVOT)^2^ [21]. It was measured at rest and during Valsalva maneuver. LVOT obstruction was defined as a systolic pressure gradient ≥30 mmHg.

### Cardiac magnetic resonance imaging (CMR) and LV chamber performance assessment

CMR was performed using a 1.5-T scanner (Magnetom Avanto®; Siemens Medical Solutions, Erlangen, Germany) with a phased array body coil. (Supplementary Method 5).

### Extent of LGE assessment

From the LGE images, the LV was divided into 16 segments [22]. Presence of LGE involvement in each segment and the total number of LGE-involving segments were measured. In addition, the percentage of LGE in LV mass were measured using dedicated quantitative analysis software (Qmass®MR 8.1, Medis, Leiden, Netherland) using LGE images with PSIR sequence. To improve the reproducibility, experienced radiologist and cardiologist with more than 10 years of experience analyzed the LGE sizes. In each short-axis slice image, the boundaries of contrast-enhanced areas were automatically traced. On LGE-MR images, myocardium with abnormal enhancement was defined as an area of hyper-enhancement more than five standard deviations from the remote myocardium. Remote myocardium was defined as non-enhanced myocardium, opposite of the hyper-enhanced myocardium [23]. The maximum signal was determined by computer-assisted window thresholding of the enhanced area. Obvious artifacts, such as those caused by motion, were excluded using a tool from the software package. Total LGE amount was calculated by summation of all slice volumes of enhancement.

### Myocardial strain analysis using feature tracking CMR

Myocardial strain analysis using feature tracking CMR was performed in 135 patients with semi-automated software (Qstrain®MR 2.0, Medis, Leiden, Netherland). LV endocardial borders were manually drawn at a reference frame. LV endocardial and epicardial borders were manually traced in 2-, 3-, 4-chamber long-axis views at end-systolic and end-diastolic phase. LV global longitudinal strain (GLS) was obtained from averaging longitudinal strains of apical 2-, 3-, and 4-chamber view. LA endocardial border was manually traced in 4-chamber long-axis view using LV end-diastole as reference phase. LA global longitudinal strain is defined as the average peak strain value. LA maximal, pre-contraction (pre-A in cases without AF) and minimal volume were measured [24]. LA total emptying fraction was calculated as (LA maximal volume – LA minimal volume) / LA maximal volume. LA reservoir fraction, as (LA maximal volume – LA minimal volume / LA minimal volume), LA conduit fraction as (LA maximal volume – LA pre-A volume) / LA maximal volume, and LA active emptying fraction as (LA pre-A volume – LA minimal volume) / LA pre-A volume [6]. (Fig. 1).

### Statistical analysis

Continuous variables with normal distributions are reported as the mean ± standard deviation or 95% confidence interval. Student’s *t*-tests were used to compare the means of continuous variables that were approximately normally distributed between the two groups. Normality was determined using the Shapiro–Wilk test. Categorical variables are reported as counts (or percentages) and were compared using chi-square tests. For comparison of more than two groups, analysis of variance was performed with post-hoc analysis (LSD) for subgroup comparison. For the correlates of LA function, Pearson’s correlation coefficients were determined and Pearson’s correlation analyses were performed. For the multivariable analysis, linear regression analysis or logistic regression analysis was performed with variables with *p* < 0.05 in univariate analysis to check the independence of the variables. All statistical analyses were performed using SPSS version 25.0 (IBM Corp., Armonk, NY, USA). A two-sided *p*-value < 0.05 was considered statistically significant.

## Results

### Baseline characteristics

The mean age of the patients was 59 ± 14 years, and 63 (30%) of them were female. Of the total patients, 49 (23%) had obstructive HCM; 100 patients (47%) had ApHCM, and 64 (64%, 64/100) of these had pure-type ApHCM. Patients with HCM had higher LA volume index and lower a’ than those of age- and sex- matched controls. CMR was performed in 135 patients (LGE in 133 patients). The mean LV mass index was 85.7 ± 22.9 g/m^2^; 92 (69%) patients showed evidence of LGE. The average number of LGE segments and %LGE mass were 3.5 ± 3.4 and 8.0 ± 9.7%, respectively (Table 1).

**Table 1:** Clinical, echocardiographic, electrocardiographic, and cardiac magnetic resonance imaging findings in controls and patients with hypertrophic cardiomyopathy according to sarcomere gene mutations

	Control (*n* = 30)	HCM (*n* = 212)		
		Presence of sarcomere protein gene variant group (n = 67)	Absence of sarcomere protein gene variant group (*n* = 145)	^**‡**^p
**Age**, years	58.9 ± 2.6	54.8 ± 14.3	61.3 ± 12.8	0.001
**Women**, n (%)	10 (33)	25 (37)	38 (26)	0.106
**Hypertension**, n (%)	11 (37)^*^	28 (42)	91 (63)	0.004
**Diabetes**, n (%)	5 (17)	12 (18)	27 (19)	0.884
**Persistent AF at echo**, n (%)		11 (16)	14 (10)	0.156
**NSVT at 24-h Holter**, n (%) (*n* = 123)		10 (22)	7 (9)	0.049
**5-year SCD risk**, % (n = 123)		2.64 ± 1.51	2.01 ± 1.66	0.040
**Echocardiography**				
**ApHCM**, n (%)		21 (31)	79 (55)	0.001
**Dynamic obstruction**, n (%)		13 (19)	36 (25)	0.370
**LV ejection fraction**, %	65.7 ± 3.8	63.8 ± 7.3	64.9 ± 5.8	0.239
**LA volume index**, mL/m^2^	19.8 ± 3.4^†^	41.0 ± 22.0	34.9 ± 15.0	0.045
**e’**, cm/s	6.9 ± 1.5^†^	5.1 ± 1.8	5.1 ± 1.7	0.960
**a’**, cm/s	10.3 ± 1.7^†^	6.9 ± 2.0	7.8 ± 1.9	0.004
**s′**, cm/s	8.8 ± 1.6^†^	6.6 ± 1.6	6.8 ± 1.8	0.408
**E**, cm/s	64.5 ± 14.1	71.0 ± 23.2	68.7 ± 18.0	0.463
**A**, cm/s	75.7 ± 15.9	61.9 ± 19.6	72.4 ± 20.8	0.001
**E/e′**	9.8 ± 2.8^†^	15.4 ± 6.7	14.6 ± 5.5	0.371
**Maximal thickness**, mm		19.7 ± 3.7	18.6 ± 3.4	0.035
**CMR** (*n* = 135)				
**LVEDV**, mL		133.6 ± 28.6	135.7 ± 28.2	0.698
**LVESV**, mL		51.7 ± 23.1	46.9 ± 18.5	0.208
**LV mass index**, g/m^2^		88.0 ± 21.1	84.8 ± 23.8	0.456
**LV mass/volume ratio**		1.19 ± 0.32	1.14 ± 0.32	0.469
**Presence of LGE in LV** (*n* = 133)		37 (90)	55 (60)	< 0.001
**Number of LGE segments in LV**(n = 133)		4.9 ± 2.8	2.9 ± 3.5	0.002
**% LGE amount of LV**(n = 133)		10.6 ± 10.1	6.4 ± 9.3	0.040
**LV myocardial GLS**, %		−14.6 ± 5.7	−16.2 ± 4.0	0.100
**LV endocardial GLS**, %		−17.5 ± 5.5	−20.6 ± 4.2	0.058
**LA minimal volume index**, mL/m^2^		49.0 ± 35.9	37.3 ± 20.4	0.053
**LA GLS**, %		17.7 ± 11.5	19.8 ± 9.1	0.288
**LA total emptying fraction**, %		37.0 ± 18.5	44.2 ± 12.4	0.025
**LA reserve fraction**, %		73.1 ± 52.1	89.3 ± 50.8	0.090
**LA conduit fraction**, % (*n* = 119)		20.0 ± 11.7	21.8 ± 8.3	0.433
**LA active emptying fraction**, % (*n* = 119)		27.0 ± 14.8	31.3 ± 11.3	0.093

*p < 0.05; ^†^*p* < 0.01, compared with total patients with hypertrophic cardiomyopathy; ^**‡**^p, comparisons between patients with and without sarcomere gene mutations; *HCM* hypertrophic cardiomyopathy; *ApHCM* apical HCM; *AF* atrial fibrillation; *NSVT* non-sustained ventricular tachycardia; *SCD* sudden cardiac death; *LV* left ventricle; *LVEDV* LV end-diastolic volume; *LVESV* LV end-systolic volume; *LVEF* LV ejection fraction; *LA* left atrial; *e’* early diastolic mitral annular velocity; *a’* late diastolic mitral annular velocity; *s’*, systolic mitral annular tissue velocity; *E* early diastolic transmitral inflow velocity; *A* late diastolic transmitral inflow velocity; *LGE* late gadolinium enhancement, *GLS* global longitudinal strain

### Genetic characteristics

Based on the American College of Medical Genetics and Genomics guidelines, [19] 67 of 212 (32%) patients had 71 pathogenic or likely pathogenic variants in 33 sarcomere-associated genes (33 *MYBPC3*, 19 *MYH7*, 14 *TNNI3*, 2 *MYH6*, 1 *JPH2*, 1 *TNNC1*, and 1 *MYL3*). Four patients harbored more than one variants in HCM genes. Homozygous or compound heterozygous variants in *MYBPC3* were identified in one patient and co-variants were identified in three patients (two had *MYBPC3* and *MYH7*, and one had *MYBPC3* and *JPH2*). In total, 26 (12%) patients had probably damaging mtDNA variants (one patient was missed due to non-analysis of mtDNA), 15 (7%) had mitochondria-related nDNA variants, and 1 had a pathogenic variant in GAA. Seven patients (3%) had both pathogenic sarcomere gene variants and mitochondria-related variants. Detailed genetic variants of detected sarcomere genes, mitochondria-related nDNAs and damaging mtDNAs are shown in Supplementary Table S2, S3 and S4 [12]. The patients with pathogenic or likely pathogenic sarcomere gene mutations (PSM) had higher prevalence of LGE (90% vs. 63% vs. 50%, *p* = 0.001) and number of LGE-involved segments (4.9 ± 2.8 vs. 2.9 ± 3.3 vs. 2.6 ± 3.8, *p* = 0.005) than patients without any mutations and those with mitochondria-related mutations.

### Effects of sarcomere mutations on LA function

**Fig. 2:**
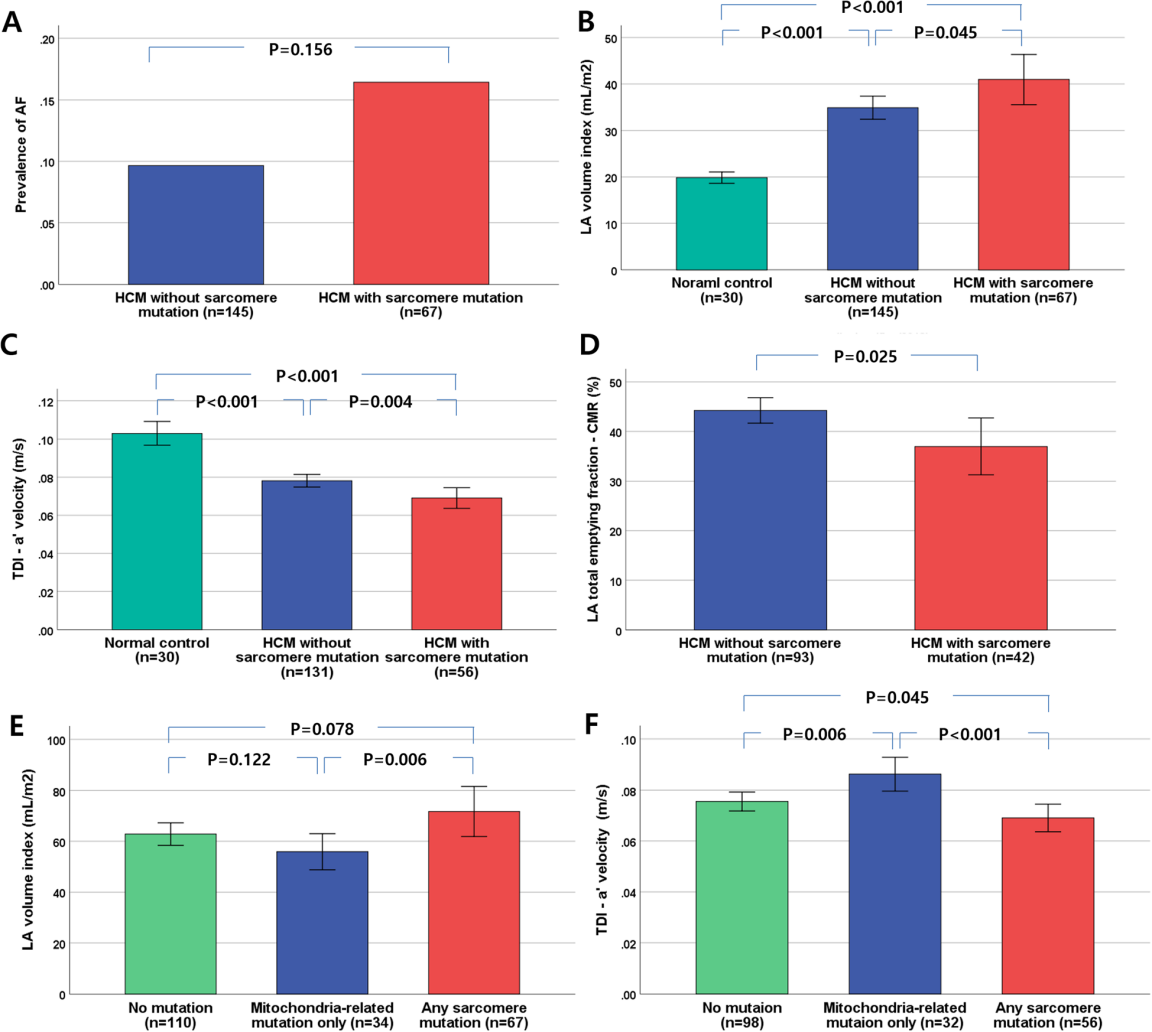
Comparison of prevalence of atrial fibrillation (**a**), left atrial volume index (**b**), and a’ velocity (**c**) and left atrial total emptying fraction (**d**) between sarcomere gene mutation-positive group and mutation-negative group. Comparison of left atrial volume index (**e**), and a’ (**f**) velocity between sarcomere- and mitochondria-related gene mutation groups^*^. HCM, hypertrophic cardiomyopathy; AF, atrial fibrillation, LA, left atrial, TDI, tissue Doppler imaging; a’, peak late-diastolic septal mitral annular; CMR, cardiac magnetic resonance imaging; bar indicates standard error; *one patient was missed due to non-analysis of mtDNA

**Fig. 3:**
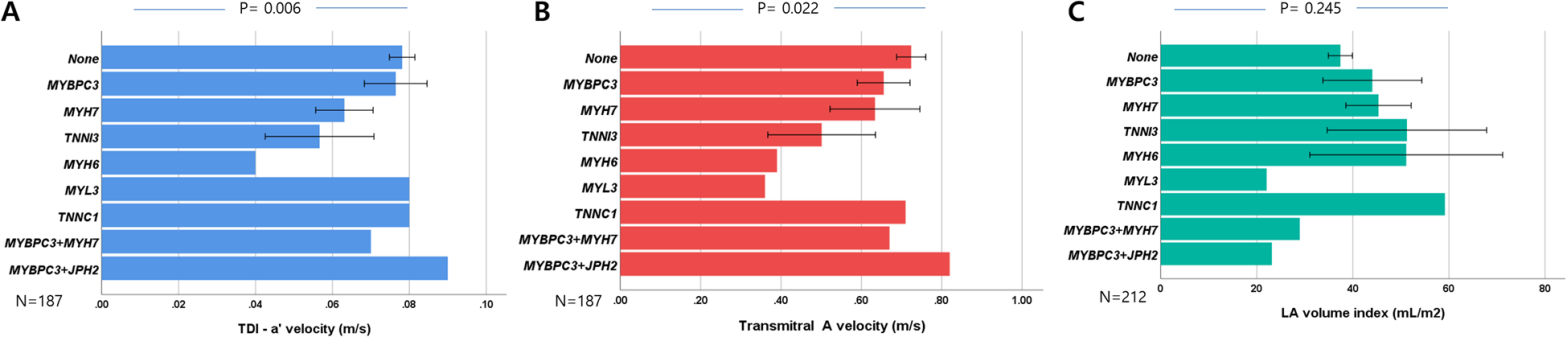
Comparisons of a’ (**a**), A (**b**) velocity and left atrial volume index (**c**) within patients with individual sarcomere gene mutations

The PSM (*n* = 67, 32%) had higher LA volume index (41.0 ± 22.0 vs. 34.9 ± 15.0 mL/m^2^, *p* = 0.045) and tendency of higher prevalence of AF (16.4% vs. 9.7%, *p* = 0.156). Even in patients without AF (*n* = 187), PSM had significantly lower A (61.9 ± 19.6 vs. 72.4 ± 20.8 cm/s, *p* = 0.001) and a’ (6.9 ± 2.0 vs. 7.8 ± 1.9 cm/s, *p* = 0.004). PSM had lower LA total emptying fraction by CMR than others (44.2 ± 12.4 vs. 37.0 ± 18.5%, *p* = 0.025). However, patients with only mitochondria-related mutations had higher a’, compared with PSM (8.6 ± 3.3 vs. 6.9 ± 2.0 cm/s, *p* < 0.001) (Fig. 2). Within PSM, those with *TNNI3* mutation and *MYH6* had lower a’, A and higher LA volume index than others (Fig. 3). Patients with thick filament mutations (*n* = 46) had a lower A (64.0 ± 19.0 vs. 71.0 ± 21.2 cm/s, *p* = 0.048) and a tendency to lower a’ (7.13 ± 1.96 vs. 7.68 ± 1.96 cm/s, *p* = 0.099) compared with those without thick filament mutations (*n* = 141).

### Correlates and independent relationship of sarcomere mutations to LA function

Patients with ApHCM had higher a’ than that of patients without ApHCM. In univariate analysis, a’ was significantly correlated to LA volume index, E/e’, e’, and s’. In addition, LV mass index, LA minimal volume, LA total emptying fraction, LA active emptying fraction, 5-year risk of sudden cardiac death, and the presence and number of LGE segments were significantly correlated to a’ but not with LV global longitudinal strain. In multivariate analysis, PSM was significantly related to a’ independent of the presence of ApHCM, E/e’, s’, and LA volume index. When using the definition of a’ < 6 cm/s (less than 3rd quartile value) as LA dysfunction, PSM was also significantly correlated to LA dysfunction independent of ApHCM, E/e’, s’ and LA volume index by echocardiography (Table 2). However, the relation significantly attenuated (*p* > 0.05) after adjustment for the presence of LGE or number of LGE segment in LV, which suggests common myopathy in LV and LA. In subgroup who underwent CMR, LA total emptying fraction was significantly correlated to age, E/e’, s’, a’, %LGE mass, LV ejection fraction by CMR, LV myocardial GLS and presence of sarcomere mutations. In multivariate analysis, presence of sarcomere mutations was significantly correlated to LA total emptying fraction independent of age, E/e’, s’, LV ejection fraction by CMR, LV myocardial GLS and %LGE mass (Table 3).

**Table 2:** Univariate and multivariate analysis for a’ velocity

	a’				LA dysfunction (a’ < 6 cm/s)			
	Univariate analysis		Multivariate analysis		Univariate analysis		Multivariate analysis	
	(r)	(p)	(β)	(p)	OR (95% CI)	p	OR (95% CI)	p
**Age**, per year	0.102	0.166			0.98 (0.95–1.01)	0.098		
**Dynamic obstruction**	−0.069	0.348			3.68 (1.60–8.45)	0.002		
**ApHCM**	0.182	0.013	0.057	0.341	0.38 (0.16–0.90)	0.029	1.34 (0.39–4.57)	0.638
**LVEF-by echo**, per %	0.030	0.683			0.93 (0.88–0.99)	0.015		
**E**, per cm/s	−0.260	< 0.001			26.39 (3.40–204.94)	0.002		
**A**, per cm/s	0.291	< 0.001			0.06 (0.006–0.49)	0.009		
**e’**, per 100 cm/s	0.232	0.001			0.74 (0.57–0.97)	0.030		
**E/e’**	0.411	< 0.001	−0.089	0.190	1.17 (1.08–1.25)	< 0.001	1.07 (0.97–1.17)	0.182
**s’**, per 100 cm/s	0.592	< 0.001	0.484	< 0.001	0.47 (0.34–0.65)	< 0.001	0.52 (0.34–0.78)	0.001
**LA volume index**, per mL/m^2^	−0.418	< 0.001	− 0.200	0.002	1.05 (1.03–1.07)	< 0.001	1.04 (1.01–1.07)	0.004
**Presence of sarcomere mutations**	0.211	0.004	−0.138	0.017	4.76 (2.05–11.02)	< 0.001	5.33 (1.71–16.61)	0.004
**LV mass index by CMR** (*n* = 135)	−0.214	0.021			1.02 (1.00–1.05)	0.046		
**Presence of LGE** (*n* = 133)	−0.248	0.007			1.89 (0.49–7.21)	0.353		
**No of LGE segment** (*n* = 133)	−0.334	< 0.001			1.26 (1.07–1.49)	0.006		
**%LGE mass** (*n* = 133)	−0.132	0.156			1.03 (0.97–1.09)	0.312		
**LV myocardial GLS** (*n* = 135)	−0.045	0.627			1.05 (0.93–1.18)	0.435		
**LA GLS** (*n* = 135), per %	0.076	0.413			0.92 (0.85–0.99)	0.034		
**LA minimal volume** (*n* = 135), per mL	−0.443	< 0.001			1.03 (1.02–1.05)	< 0.001		
**LA maximal volume** (*n* = 135), per mL	−0.427	< 0.001			1.02 (1.01–1.04)	0.001		
**LA total emptying fraction** (*n* = 135), per %	0.251	0.006			0.93 (0.89–0.97)	0.001		
**LA active emptying fraction** (*n* = 119), per %	0.248	0.006			0.93 (0.88–0.97)	0.003		
**5-yr-SCD risk** (*n* = 123)	−0.314	0.001			1.45 (1.06–1.99)	0.020		
**Presence of NSVT** (*n* = 123)	−0.214	0.036			5.27 (1.58–17.66)	0.007		

Abbreviations are same as in Table 1

**Table 3:** Univariate and multivariate analysis for left atrial total emptying fraction

LA total emptying fraction (%) by CMR	Univariate analysis		Multivariate analysis	
	(r)	(p)	(β)	(p)
**Age**, per year	−0.334	< 0.001	−0.265	< 0.001
**Dynamic obstruction**	0.163	0.069		
**ApHCM**	0.148	0.089		
**E/e’**	−0.178	0.038	− 0.052	0.497
**s’**, per cm/s	0.370	< 0.001	0.164	0.043
**a’**, per cm/s^a^	0.251	0.006		
**Presence of sarcomere mutations**	−0.226	0.008	− 0.171	0.015
**LV mass index by CMR**, g/m^2^	−0.040	0.647		
**Presence of LGE** (*n* = 133)	−0.086	0.218		
**No of LGE segment** (*n* = 133)	−0.172	0.048		
**%LGE mass** (*n* = 133)	−0.182	0.036	0.042	0.566
**LVEF by CMR**, per %	0.274	0.001	0.103	0.186
**LV myocardial GLS**, per %	−0.537	< 0.001	−0.429	< 0.001
**LA GLS**, per %^a^	0.824	< 0.001		
**LA minimal volume**, per mL^a^	−0.677	< 0.001		
**LA maximal volume**, per mL^a^	−0.408	< 0.001		
**LA active emptying fraction** (*n* = 119), per %^a^	0.879	< 0.001		

^a^Due to collinearity to LA total emptying fraction, they were not included in the multivariate analysis. See abbreviations in Table 1

## Discussion

This study shows the following major findings. First, LA function was significantly reduced in patients with HCM, compared with controls despite same age and sex distribution. Second, among patients with HCM, the PSM had significantly reduced LA function (measured by a’ in echo and LA total emptying fraction by CMR), compared with patients without sarcomere gene mutations. Third, worse LA function in the PSM was independent of the LA volume index, LV mass index, and E/e’. Fourth, LA function, measured by a’, was significantly correlated to the extent of LGE in LV, and the PSM had significantly higher extent of LGE. Although, the relationship between sarcomere gene mutation and a’ was significantly attenuated after adjustment for the extent of LGE in LV, the LA total emptying fraction was significantly correlated to sarcomere gene mutation independent of %LGE mass, which suggests that LA dysfunction is a unique finding of HCM as an LA myopathy related to sarcomere gene mutations independent of LA loading conditions. Although we did not measure LA fibrosis by LGE quantification, this finding suggests that sarcomere gene mutation may contribute to both LV and LA myopathy, and may then induce LA dysfunction. This sarcomere gene mutation- related LA dysfunction is not just through elevated LV filling pressure, because even after adjustment for E/e’, LA volume index, and LV mass index, sarcomere gene mutation-related LA dysfunction remained significant. In addition, the findings that PSM were significantly younger than those in the non-sarcomere-related group and the LA function of patients with HCM was significantly lower than controls despite same age and sex distribution, suggest that LA dysfunction in HCM is a unique myopathic process and that sarcomere gene mutation significantly contribute to LA dysfunction. Previous basic studies showed that sarcomere gene mutation directly induces myocyte hypertrophy, myocyte disarray, and fibrosis in the LV in HCM mouse models [25]. Some studies showed that TGF β1-medicated LA fibrosis also developed in sarcomere gene mutation-derived mouse HCM models only with dysfunctional LV [25], which supports our speculation of a common myopathic process in the LV and LA. This finding also suggests that current anti-hypertrophy and anti-fibrotic agents [26] would affect LA structural and functional remodeling, thereby reducing AF. In our study, mitochondria-related mutations were not significantly related to LA dysfunction and LV fibrosis; therefore, phenotypic contribution and myocardial fibrosis is mainly contributed by pathogenic sarcomere gene mutations. Despite the small number of subjects with validated pathogenic sarcomere gene mutations in our study, the degree of genetic contribution to LA function seemed to vary between mutations. According to our study, patients with *TNNI3* mutation had worse LA function than patients with *MYBPC3* mutation did, whereas mitochondria-related gene mutations showed more benign phenotypes in terms of LA function. Therefore, gene-targeted therapy needs to be individualized.

Prevention of sudden cardiac death due to ventricular tachycardia is a major goal of management of HCM [27]. However, several recent studies showed that the general prognosis of HCM is better than that in previous reports, and active primary prevention has reduced the rate of sudden deaths related to HCM [28]. With the advancing age of patients with HCM, heart failure due to advanced diastolic dysfunction or development of AF become a major concern [29]. In fact, several studies showed that concomitant or newly developed AF is a major prognostic factor for heart failure admission, new-onset stroke, and mortality in HCM [30, 31]. Therefore, prevention of AF should be one of the goals of management of HCM. In this regard, several new attempts for upstream genetic modulation using CRISPR/Cas9, [32] a sarcomere-targeted anti-hypertrophic or anti-fibrotic agent, [26] could have potential preventive effects on the development of AF and heart failure.

This study has several limitations. First, we did not evaluate the myopathic process of the LA itself, such as direct tissue characterization or CMR-derived LA fibrosis measurement [33]. However, measurement of LA-LGE involves extensive variability, and 3D-LGE is not routinely measured in most institutions. Hence, use of the most popular parameters with less measurement variability would be justified. Further studies are warranted to develop a more accurate and reproducible method for assessing the LA myopathic process in HCM. Second, owing to lack of long-term follow-up data, the relationship between LA dysfunction and future development of new-onset AF or heart failure was not proved. However, several previous studies showed that LA structural or functional remodeling was significantly related to poor outcomes, especially to the development of new-onset AF and heart failure [1, 2]. The findings of this study could help elucidate this relationship. In addition, our results attempt to explain the reason behind the worse prognosis of PSM among those with HCM. Third, due to limited number of CMR, LA active emptying fraction did not reach the statistical differences between sarcomere groups. However, LA total emptying fraction was significantly lower in sarcomere mutation groups even in the limited patients, which supports our observation measured by a’ velocity. Larger number of echocardiographic or CMR studies with phasic LA functional analyses warrant further investigation.

## Conclusion

Presence of pathogenic sarcomere mutation was significantly related to LA dysfunction independent of LV filling pressure and LAVI, suggesting its contribution to atrial myopathy in HCM. Therefore, our study results may provide an explanation why HCM patients with sarcomere gene mutations had poorer prognosis, regarding higher prevalence of AF and hospitalization due to heart failure [34].

## Supplementary Information

**Additional file 1: Method S1.** DNA preparation. **Method S2.** Library construction and sequencing of the HCM gene panel. **Method S3**. Library construction and mtDNA sequencing. **Method S4.** Data analysis of the mitochondrial genome. **Method S5**. CMR. **Table S1.** Summary of 82 genes associated with hypertrophic cardiomyopathy. **Supplementary Table S2.** Likely pathogenic or Pathogenic variants in the 33 sarcomere associated genes classified according to the refined American College of Medical Genetics and Genomics (ACMG) standards and guidelines for inherited cardiac conditions. **Supplementary Table S3.** Likely pathogenic or pathogenic variants in the 6 non-sarcomere genes and the 44 mitochondria-related nuclear genes. **Supplementary Table S4.** Non-haplogroup-associated variants with a GenBank frequency < 0.1%.

## Data Availability

The datasets of the current study are available from the corresponding author on reasonable request.

## Abbreviations

LA: Left atrial
AF: Atrial fibrillation
HCM: Hypertrophic cardiomyopathy
a’: Tissue Doppler imaging-based late mitral annular velocity
LV: Left ventricular
ApHCM: Apical hypertrophic cardiomyopathy
nDNA: Nuclear DNA
mtDNA: Mitochondrial DNA
CMR: Cardiac magnetic resonance imaging
LGE: Late gadolinium enhancement
PSM: Patients with pathogenic or likely pathogenic sarcomere gene mutations
E: Early diastolic transmitral inflow velocity
s’: Tissue Doppler imaging-based systolic mitral annular tissue velocity
GLS: Global longitudinal strain
