# Supplementary material for: Contribution of sarcomere gene mutations to left atrial function in patients with hypertrophic cardiomyopathy
Source: Cardiovasc Ultrasound. 2021 Jan 6;19:4. doi: 10.1186/s12947-020-00233-y (PMC7789152; doi:10.1186/s12947-020-00233-y)
Supplement: Supplementary file 1 — Additional file 1: Method S1. DNA preparation. Method S2. Library construction and sequencing of the HCM gene panel. Method S3. Library construction and mtDNA sequencing. Method S4. Data analysis of the mitochondrial genome. Method S5. CMR. Table S1. Summary of 82 genes associated with hypertrophic cardiomyopathy. Supplementary Table S2. Likely pathogenic or Pathogenic variants in the 33 sarcomere associated genes classified according to the refined American College of Medical Genetics and Genomics (ACMG) standards and guidelines for inherited cardiac conditions. Supplementary Table S3. Likely pathogenic or pathogenic variants in the 6 non-sarcomere genes and the 44 mitochondria-related nuclear genes. Supplementary Table S4. Non-haplogroup-associated variants with a GenBank frequency < 0.1%. [file 12947_2020_233_MOESM1_ESM.docx]

**Method S1. DNA preparation**

Genomic DNA was extracted from EDTA-treated whole blood samples by using a QIAamp DNA Blood Mini kit (Qiagen, Hilden, Germany) on a QIAcube automatic nucleic acid extrac­tion instrument (Qiagen) according to the manufacturer’s in­structions. The DNA samples were used to analyze mtDNA and the HCM gene panel (nDNA).

**Method S2. Library construction and sequencing of the HCM gene panel**

For targeted sequencing, DNA fragments were enriched by solution-based hybridization capture and sequenced on an Illumina Hiseq2500 platform (Illumina, San Diego, CA, USA) with the 2 × 150 base pair paired-end read module. Genomic DNA was sheared using an Adaptive Focused Acoustics™ with a Covaris Focused-ultrasonicator (Covaris, Inc., Woburn, MA, USA). The quality and quantity of sheared DNA were assessed using the Agilent 2200 Tape Station system with Agilent D1000 ScreenTape (Agilent Technologies, Santa Clara, CA, USA) according to the manufacturer’s instructions. Capture probes were customized and produced by Celemics, Inc. (Seoul, Korea) to cover coding sequence regions of 82 target genes. Purification and clean-up of samples were also performed with AMPure beads. NGS library amplification was performed using a KAPA Library Amplification Kit (Kapa Biosystems, Inc., Wilmington, MA, USA) according to the manufacturer’s instructions. Library preparation, hybridization, capture procedure, and sequencing were performed by Celemics according to the protocols recommended by the Celemics User Manual Ver 2.1 (<http://www.celemics.com/home/>).

**Method S3. Library construction and mtDNA sequencing**

The complete mtDNA was amplified by using four overlapping pairs of primers.[1] Library preparations were performed following the manufacturer’s instructions (Ion XpressPlus Fragment Library Kit; Thermo Fisher Scientific, Waltham, MA, USA) for 400 single-end reads. Library material was purified using AMPure beads (Beckman Coulter, Brea, CA, USA). For multiplexing of the samples, each DNA library was barcoded using different ligation adaptors. The fragmented and adaptor ligated libraries were selected following electrophoretic separation with the E-gel SizeSelect gel (Thermo Fisher Scientific) following the manufacturer’s recommendations. Subsequent emulsion polymerase chain reaction and enrichment of the sequencing beads of the pooled libraries was performed using the OneTouch system (Thermo Fisher Scientific) according to the manufacturer’s protocol. Finally, sequencing was conducted on the 318 chip using Ion PGM Hi-Q Sequencing Kit on the Ion Torrent Personal Genome Machine (Thermo Fisher Scientific).

**Method S4.** **Data analysis of the mitochondrial genome**

Quality-filtered sequences were aligned to the Revised Cambridge Reference Sequence (rCRS; NC_012920) and analyzed.[2] Basic data analysis was performed using Torrent Suite Software version 5.2.1 with the default parameters (Life Technologies, Carlsbad, CA, USA) and the plug-in VariantCaller for mtDNA employing TMAP Smith–Waterman alignment optimization.[3] The output of the variant caller was presented in a tabular format as a list of variations to the revised Cambridge Reference (rCRS) with total coverage and variant frequency values. Variant detection was called to detect insertions and deletions as well as single-nucleotide polymorphisms with reference to the rCRS. Visual inspection of the mapped data was performed using Integrated Genomics Viewer 2.3 software (IGV; Broad Institute, Cambridge, MA, USA). Mitochondrial genome databases, including MITOMAP[4] and Human Mitochondrial Genome Database (mtDB)[5] and Phylotree[6] were referred to validate the detected variants. Novel and rare non-haplogroup-associated variants were further evaluated for their potential pathogenicity based on the variant’s location, changes in the amino acid sequence, and evolutionary conservation.[7] We have assessed potential pathogenicity using multiple software programs including Polyphen2, Fathmmw, Mutation Assessor, and PROVEAN. When the majority of computational evidence supported a deleterious effect, we have assigned novel and rare non-haplogroup-associated variants as damaging mtDNA variants.

**Method S5. CMR**

The LV 2-, 3-, 4-chamber, and short axis views were obtained using cine images with steady-state free precession sequence.[8] After administration of 0.2 mmol/kg of a gadolinium-based contrast agent (gadoterate dimeglumine; Dotarem, Guerbet, France), late gadolinium enhancement (LGE) imaging, with a magnitude- and phase-sensitive inversion recovery-prepared fast gradient echo sequence (PSIR), was performed in 10 minutes. A bolus of contrast media was intravenously administered at 2 mL/s, followed by 20 mL of normal saline at 4 mL/s through a 20-gauge cannula in the antecubital vein using a power injector (Nemoto; Nemoto Kyorindo, Tokyo, Japan). The appropriate inversion time before LGE imaging was determined using a fast gradient echo sequence with inversion times varying from 150 to 650 ms to null the signal from the normal myocardium. The LGE imaging parameters were as follows: TR = 8.8 ms, TE = 3.36 ms, flip angle = 25°, acquisition matrix = 256 × 166, and field of view = 276 × 340 mm.

***LV geometry and chamber performance assessment***

The endocardial and epicardial borders were contoured using a semi-automated method (Argus®; Siemens, Germany or Qmass® MR 8.1; Medis, Leiden, the Netherlands); subsequently, the LVEDV and LVESV were measured.[9] To determine the end-diastolic LV mass, the difference between the epicardial and endocardial areas for all slices was multiplied by the slice thickness and section gap, and then multiplied by the specific gravity of the myocardium (1.05 g/mL). Papillary muscle mass was included in the LV cavity and excluded from the LV mass measurements. Stroke volume was calculated as LVEDV minus LVESV, and LV ejection fraction was calculated as 100 multiplied by the stroke volume and then divided by LVEDV. LV mass index was calculated by dividing the LV mass by the BSA.

| **Table S1.** Summary of 82 genes associated with hypertrophic cardiomyopathy | | | | | | |
| --- | --- | --- | --- | --- | --- | --- |
| Category | Gene | OMIM number | Transcript reference | Phenotype | Inheritance | Reference |
| Sarcomere associated genes (validated) | ACTC1 | * 102540 | NM_005159.4 | Cardiomyopathy, hypertrophic, 11 | AD | [10] |
|  | MYBPC3 | * 600958 | NM_000256.3 | Cardiomyopathy, hypertrophic, 4 | AD | [10] |
|  | MYH7 | * 160760 | NM_000257.3 | Cardiomyopathy, hypertrophic, 1 | AD | [10] |
|  | MYL2 | * 160781 | NM_000432.3 | Cardiomyopathy, hypertrophic, 1 | AD | [10] |
|  | MYL3 | * 160790 | NM_000258.2 | Cardiomyopathy, hypertrophic, 8 | AD | [10] |
|  | TNNI3 | * 191044 | NM_000363.4 | Cardiomyopathy, hypertrophic, 7 | AD | [10] |
|  | TNNT2 | * 191045 | NM_001001430.2 | Cardiomyopathy, hypertrophic, 2 | AD | [10] |
|  | TPM1 | * 191010 | NM_001018005.1 | Cardiomyopathy, hypertrophic, 3 | AD | [10] |
| Sarcomere associated genes (putative) | CSRP3 | * 600824 | NM_003476.4 | Cardiomyopathy, hypertrophic, 12 | AD | [10] |
|  | FHL1 | * 300163 | NM_001449.4 | Emery-Dreifuss muscular dystrophy 6, X-linked | XLR | [10] |
|  | PLN | * 172405 | NM_002667.3 | Cardiomyopathy, hypertrophic, 18 | AD | [10] |
|  | ACTN2 | * 102573 | NM_001103.3 | Cardiomyopathy, hypertrophic, 23, with or without LVNC | AD | [10] |
|  | CRYAB | * 123590 | NM_001885.1 | Cardiomyopathy, dilated, 1II | AD | [10] |
|  | MYOZ2 | * 605602 | NM_016599.4 | Cardiomyopathy, hypertrophic, 16 | AD | [10] |
|  | MYH6 | * 160710 | NM_002471.3 | Cardiomyopathy, hypertrophic, 14 | AD | [10] |
|  | TNNC1 | * 191040 | NM_003280.2 | Cardiomyopathy, hypertrophic, 13 | AD | [10] |
|  | ANKRD1 | * 609599 | NM_014391.2 | Hypertrophic Cardiomyopathy | Not evaluated | [10] |
|  | CAV3 | * 601253 | NM_033337.2 | Cardiomyopathy, familial hypertrophic | AD | [10] |
|  | JPH2 | * 605267 | NM_020433.4 | Cardiomyopathy, hypertrophic, 17 | AD | [10] |
|  | LDB3 | * 605906 | NM_007078.2 | Cardiomyopathy, hypertrophic, 24 | AD | [10] |
|  | MYLK2 | * 606566 | NM_033118.3 | Cardiomyopathy, hypertrophic, 1, digenic | AD | [10] |
|  | MYOM1 | * 603508 | NM_003803.3 | Hypertrophic Cardiomyopathy | Not evaluated | [10] |
|  | MYPN | * 608517 | NM_032578.3 | Cardiomyopathy, hypertrophic, 22 | AD | [10] |
|  | NEXN | * 613121 | NM_144573.3 | Cardiomyopathy, hypertrophic, 2 | AD | [10] |
|  | TCAP | * 604488 | NM_003673.3 | Cardiomyopathy, hypertrophic, 25 | AD | [10] |
|  | BAG3 | * 603883 | NM_004281.3 | Cardiomyopathy, dilated, 1HH | AD | [11] |
|  | CASQ2 | * 114251 | NM_001232.3 | Ventricular tachycardia, catecholaminergic polymorphic, 2 | AR | [10] |
|  | MYO6 | * 600970 | NM_004999.3 | Deafness, autosomal dominant 22, with hypertrophic cardiomyopathy | AD | [10] |
|  | OBSCN | * 608616 | NM_052843.3 | Inherited cardiomyopathies | Not evaluated | [10] |
|  | RYR2 | * 180902 | NM_001035.2 | Arrhythmogenic right ventricular dysplasia 2 | AD | [12] |
|  | TNNC2 | * 191039 | NM_003279.2 | Cardiomyopathy | Not evaluated | [13] |
|  | TTN | * 188840 | NM_001267550.1 | Cardiomyopathy, familial hypertrophic, 9 | AD/AR | [14] |
|  | VCL | * 193065 | NM_014000.2 | Cardiomyopathy, hypertrophic, 15 | AD | [10] |
| Phenocopies genes | GAA | * 606800 | NM_001079803.2 | Pompe disease | AR | [15] |
|  | LAMP2 | * 309060 | NM_002294.2 | Danon disease | XLD | [15] |
|  | PRKAG2 | * 602743 | NM_016203.3 | Wolff-Parkinson-White syndrome, conduction disease | AD | [15] |
|  | PTPN11 | * 176876 | NM_002834.3 | LEOPARD syndrome, Noonan syndrome | AD | [15] |
|  | TTR | * 176300 | NM_000371.3 | Transthyretin amyloidosis | AD | [16] |
| Mitochondria- related nuclear DNA genes | AARS2 | * 612035 | NM_020745.3 | Combined oxidative phosphorylation deficiency 8 | AR | [17] |
|  | ACAD9 | * 611103 | NM_014049.4 | Mitochondrial complex I deficiency due to ACAD9 deficiency | AR | [18] |
|  | ACADVL | * 609575 | NM_000018.3 | VLCAD deficiency | AR | [19] |
|  | AGK | * 610345 | NM_018238.3 | Sengers Syndrome | AR | [20] |
|  | COA5 | * 613920 | NM_001008215.1 | Cardioencephalomyopathy, fatal infantile, due to cytochrome c oxidase deficiency 3 | AR | [21] |
|  | COA6 | * 614772 | NM_001206641.2 | Cardioencephalomyopathy, fatal infantile, due to cytochrome c oxidase deficiency 4 | AR | [22] |
|  | COQ2 | * 609825 | NM_015697.7 | Coenzyme Q10 deficiency, primary, 1 | AR | [23] |
|  | COQ4 | * 612898 | NM_016035.3 | Coenzyme Q10 deficiency, primary, 7 | AR | [23] |
|  | COQ9 | * 612837 | NM_020312.3 | Coenzyme Q10 deficiency, primary, 5 | AR | [23] |
|  | COX10 | * 602125 | NM_001303.3 | Leigh syndrome due to mitochondrial COX4 deficiency | AR | [24] |
|  | COX14 | * 614478 | NM_032901.2 | Mitochondrial complex IV deficiency | AR | [25] |
|  | COX15 | * 603646 | NM_004376.5 | Leigh syndrome due to cytochrome c oxidase deficiency | AR | [26] |
|  | COX6B1 | * 124089 | NM_001863.4 | Mitochondrial complex IV deficiency | AR | [27] |
|  | CPT2 | * 600650 | NM_000098.2 | CPT II deficiency | AR | [28] |
|  | ECHS1 | * 602292 | NM_004092.3 | Mitochondrial short-chain enoyl-CoA hydratase 1 deficiency | AR | [29] |
|  | ELAC2 | * 605367 | NM_018127.6 | Combined oxidative phosphorylation deficiency 17 | AR | [30] |
|  | FOXRED1 | * 613622 | NM_017547.3 | Leigh syndrome due to mitochondrial complex I deficiency | AR | [31] |
|  | GTPBP3 | * 608536 | NM_032620.3 | Combined oxidative phosphorylation deficiency 23 | AR | [32] |
|  | HADHB | * 143450 | NM_000183.2 | Trifunctional protein deficiency | AR | [33] |
|  | LRPPRC | * 607544 | NM_133259.3 | Leigh syndrome, French-Canadian type | AR | [34] |
|  | MRPL3 | * 607118 | NM_007208.3 | Combined oxidative phosphorylation deficiency 9 | AR | [35] |
|  | MRPL44 | * 611849 | NM_022915.3 | Combined oxidative phosphorylation deficiency 16 | AR | [36] |
|  | MRPS22 | * 605810 | NM_020191.2 | Combined oxidative phosphorylation deficiency 5 | AR | [26] |
|  | MTO1 | * 614667 | NM_012123.3 | Combined oxidative phosphorylation deficiency 10 | AR | [37] |
|  | NDUFA10 | * 603835 | NM_004544.3 | Leigh syndrome | AR | [38] |
|  | NDUFA11 | * 612638 | NM_175614.4 | Mitochondrial complex I deficiency | AR | [39] |
|  | NDUFA2 | * 602137 | NM_002488.4 | Leigh syndrome due to mitochondrial complex I deficiency | AR | [26] |
|  | NDUFAF1 | * 606934 | NM_016013.2 | Mitochondrial complex I deficiency | AR | [40] |
|  | NDUFS2 | * 602985 | NM_004550.4 | Mitochondrial complex I deficiency | AR | [41] |
|  | NDUFS4 | * 602694 | NM_002495.2 | Mitochondrial complex I deficiency | AR | [42] |
|  | NDUFS8 | * 602141 | NM_002496.3 | Leigh syndrome due to mitochondrial complex I deficiency | AR | [43] |
|  | NDUFV2 | * 600532 | NM_021074.4 | Mitochondrial complex I deficiency | AR | [26] |
|  | PCCB | * 232050 | NM_000532.4 | Propionicacidemia | AR | [44] |
|  | SCO2 | * 604272 | NM_005138.2 | Cardioencephalomyopathy, fatal infantile, due to cytochrome c oxidase deficiency 1 | AR | [26] |
|  | SDHD | * 602690 | NM_003002.2 | Mitochondrial complex II deficiency | AR | [45] |
|  | SLC22A5 | * 603377 | NM_003060.3 | Carnitine deficiency, systemic primary | AR | [46] |
|  | SLC25A20 | * 613698 | NM_000387.5 | Carnitine-acylcarnitine translocase deficiency | AR | [47] |
|  | SLC25A3 | * 600370 | NM_005888.3 | Mitochondrial phosphate carrier deficiency | Not evaluated | [26] |
|  | SLC25A4 | * 103220 | NM_001151.3 | Mitochondrial DNA depletion syndrome 12A /12B | AD/AR | [48] |
|  | SURF1 | * 185620 | NM_003172.2 | Leigh syndrome, due to COX IV deficiency | AR | [49] |
|  | TMEM70 | * 612418 | NM_017866.5 | Mitochondrial complex V (ATP synthase) deficiency, nuclear type 2 | AR | [26] |
|  | TRMT5 | * 611023 | NM_020810.3 | Combined oxidative phosphorylation deficiency 26 | AR | [50] |
|  | TSFM | * 604723 | NM_001172696.1 | Combined oxidative phosphorylation deficiency 3 | AR | [26] |
|  | YARS2 | * 610957 | NM_001040436.2 | Myopathy, lactic acidosis, and sideroblastic anemia 2 | AR | [51] |

**Supplementary Table S2.** Likely pathogenic or Pathogenic variants in the 33 sarcomere associated genes classified according to the refined American College of Medical Genetics and Genomics (ACMG) standards and guidelines for inherited cardiac conditions

| Case | Gene | Reference sequence | DNA Change | Amino Acid Change | Clinvar accession | Clinvar variant class | HGMD accession | HGMD phenotype | HGMD variant class | MAF (ExAC) | MAF (KRGDB) | dbSNP |
| --- | --- | --- | --- | --- | --- | --- | --- | --- | --- | --- | --- | --- |
| YMC-2 | *TNNI3* | NM_000363.4 | c.434G>A | p.Arg145Gln | RCV000208273.1 / RCV000159223.4 | LP / P | CM971497 | HCM | DM | 0.00002490 | 0.000909 | rs397516349 |
| YMC-3 | *MYH7* | NM_000257.3 | c.1324C>T | p.Arg442Cys | RCV000162335.1 / RCV000464365.1 | LP / P | CM066924 | HCM | DM | 0.00000824 | 0.00000000 | rs148808089 |
| YMC-4 | *MYH7* | NM_000257.3 | c.1324C>T | p.Arg442Cys | RCV000162335.1 / RCV000464365.1 | LP / P | CM066924 | HCM | DM | 0.00000824 | 0.00000000 | rs148808089 |
| YMC-7 | *MYBPC3* | NM_000256.3 | c.1484G>A | p.Arg495Gln | RCV000168090.5 | LP / P | CM981324 | HCM | DM | 0.00000829 | 0.00000000 | rs200411226 |
| YMC-15 | *TNNI3* | NM_000363.4 | c.434G>A | p.Arg145Gln | RCV000208273.1 / RCV000159223.4 | LP / P | CM971497 | HCM | DM | 0.00002490 | 0.000909 | rs397516349 |
| YMC-17 | *MYBPC3* | NM_000256.3 | c.2512G>T | p.Glu838* | (-) | (-) | CM1313299 | HCM | DM | 0.00000000 | 0.00000000 | Novel |
| YMC-20 | *MYH7* | NM_000257.3 | c.4130C>T | p.Thr1377Met | RCV000035886.3/RCV000208315.1 | VUS / LP | CM034558 | HCM | DM | 0.00000000 | 0.000455 | rs397516201 |
| YMC-25 | *MYBPC3* | NM_000256.3 | c.1000G>A | p.Glu334Lys | RCV000168764.2 | LP | CM073211 | HCM | DM | 0.00033786 | 0.006364 | rs573916965 |
| YMC-27 | *TNNI3* | NM_000363.4 | c.434G>A | p.Arg145Gln | RCV000208273.1 / RCV000159223.4 | LP / P | CM971497 | HCM | DM | 0.00002490 | 0.000909 | rs397516349 |
| YMC-30 | *TNNI3* | NM_000363.4 | c.434G>A | p.Arg145Gln | RCV000208273.1 / RCV000159223.4 | LP / P | CM971497 | HCM | DM | 0.00002490 | 0.000909 | rs397516349 |
| YMC-32 | *MYBPC3* | NM_000256.3 | c.86delT | p.Phe29Serfs*10 | (-) | (-) | (-) | (-) | (-) | 0.00000000 | 0.00000000 | Novel |
| YMC-37 | *MYBPC3* | NM_000256.3 | c.2459G>A | p.Arg820Gln | RCV000158159.3/RCV000009148.6 | LP | CM034547 | HCM | DM | 0.00001657 | 0.00000000 | rs2856655 |
|  | *MYH7* | NM_000257.3 | c.1324C>T | p.Arg442Cys | RCV000162335.1 / RCV000464365.1 | LP / P | CM066924 | HCM | DM | 0.00000824 | 0.00000000 | rs148808089 |
| YMC-40 | *MYH7* | NM_000257.3 | c.4130C>T | p.Thr1377Met | RCV000035886.3/RCV000208315.1 | VUS / LP | CM034558 | HCM | DM | 0.00000000 | 0.000455 | rs397516201 |
| YMC-41 | *MYBPC3* | NM_000256.3 | c.2833_2834del | p.Arg945Glyfs | RCV000035530.2 | P | CD022562 | HCM | DM | 0.00000000 | 0.00000000 | rs397515987 |
| YMC-43 | *MYBPC3* | NM_000256.3 | c.1505G>A | p.Arg502Gln | RCV000168303.2 | P | CM981325 | HCM | DM | 0.00000000 | 0.00000000 | rs397515907 |
|  | *JPH2* | NM_020433.4 | c.1037_1047del | p.Leu346Hisfs*50 | (-) | (-) | (-) | (-) | (-) | 0.00000000 | 0.00000000 | Novel |
| YMC-51 | *MYBPC3* | NM_000256.3 | c.2067+1G>A | (-) | (-) | (-) | CS063340 | HCM | DM | 0.00000000 | 0.00160800 | (-) |
| YMC-52 | *MYH7* | NM_000257.3 | c.599C>T | p.Ala200Val | (-) | (-) | (-) | (-) | (-) | 0.00000000 | 0.00000000 | Novel |
| YMC-54 | *TNNI3* | NM_000363.4 | c.485G>C | p.Arg162Pro | RCV000036301.3 | LP | CM031380 | HCM | DM | 0.00000000 | 0.00000000 | rs397516354 |
| YMC-55 | *MYBPC3* | NM_000256.3 | c.2272G>A | p.Gly758Ser | (-) | (-) | (-) | (-) | (-) | 0.00000000 | 0.00000000 | Novel |
| YMC-60 | *MYBPC3* | NM_000256.3 | c.3805G>T | p.Glu1269* | (-) | (-) | (-) | (-) | (-) | 0.00000000 | 0.00000000 | Novel |
|  | *MYBPC3* | NM_000256.3 | c.1000G>A | p.Glu334Lys | RCV000168764.2 | LP | CM073211 | HCM | DM | 0.00033786 | 0.006364 | rs573916965 |
| YMC-61 | *MYH7* | NM_000257.3 | c.2608C>T | p.Arg870Cys | RCV000148707.1 / RCV000158554.1 | LP | CM003687 | HCM | DM | 0.00000824 | 0.000804 | rs138049878 |
| YMC-73 | *MYH7* | NM_000257.3 | c.1350G>T | p.Lys450Asn | (-) | (-) | (-) | (-) | (-) | 0.00000000 | 0.00000000 | Novel |
| YMC-74 | *MYBPC3* | NM_000256.3 | c.2441_2443del | p.Lys814del | RCV000157327.3 | VUS / LP | CD021840 | HCM | DM | 0.00000000 | 0.00000000 | rs727504288 |
| YMC-78 | *MYH7* | NM_000257.3 | c.4130C>T | p.Thr1377Met | RCV000035886.3/RCV000208315.1 | VUS / LP | CM034558 | HCM | DM | 0.00000000 | 0.000455 | rs397516201 |
| YMC-79 | *TNNC1* | NM_003280.2 | c.23C>T | p.Ala8Val | RCV000037762.3 / RCV000159204.2 / RCV000013256.23 | VUS / LP / P | CM083571 | HCM | DM | 0.00000000 | 0.00000000 | rs267607125 |
| YMC-80 | *MYH7* | NM_000257.3 | c.2606G>C | p.Arg869Pro | (-) | (-) | (-) | (-) | (-) | 0.00000000 | 0.00000000 | Novel |
| YMC-83 | *TNNI3* | NM_000363.4 | c.433C>G | p.Arg145Gly | RCV000441050.1 / RCV000013231.25 | P | CM971498 | HCM | DM | 0.00000000 | 0.00000000 | rs104894724 |
| YMC-85 | *MYBPC3* | NM_000256.3 | c.2067+1G>A | (-) | (-) | (-) | CS063340 | HCM | DM | 0.00000000 | 0.00160800 | (-) |
| YMC-88 | *MYBPC3* | NM_000256.3 | c.3490+1G>A | (-) | RCV000009137.5 | P | CS971817 | HCM | DM | 0.00000000 | 0.00000000 | rs397516020 |
| YMC-89 | *MYBPC3* | NM_000256.3 | c.178G>T | p.Glu60* | (-) | (-) | (-) | (-) | (-) | 0.00000000 | 0.00000000 | Novel |
| YMC-90 | *MYBPC3* | NM_000256.3 | c.2833_2834del | p.Arg945Glyfs | RCV000035530.2 | P | CD022562 | HCM | DM | 0.00000000 | 0.00000000 | rs397515987 |
| YMC-93 | *MYH7* | NM_000257.3 | c.4130C>T | p.Thr1377Met | RCV000035886.3/RCV000208315.1 | VUS / LP | CM034558 | HCM | DM | 0.00000000 | 0.000455 | rs397516201 |
| YMC-94 | *TNNI3* | NM_000363.4 | c.434G>A | p.Arg145Gln | RCV000208273.1 / RCV000159223.4 | LP / P | CM971497 | HCM | DM | 0.00002490 | 0.000909 | rs397516349 |
| YMC-95 | *TNNI3* | NM_000363.4 | c.434G>A | p.Arg145Gln | RCV000208273.1 / RCV000159223.4 | LP / P | CM971497 | HCM | DM | 0.00002490 | 0.000909 | rs397516349 |
| YMC-96 | *MYBPC3* | NM_000256.3 | c.2441_2443del | p.Lys814del | RCV000157327.3 | VUS / LP | CD021840 | HCM | DM | 0.00000000 | 0.00000000 | rs727504288 |
| YMC-104 | *MYBPC3* | NM_000256.3 | c.3763delG | p.Ala1255Profs*76 | RCV000168826.2 | LP | (-) | (-) | (-) | 0.00000000 | 0.00000000 | rs786204362 |
| YMC-108 | *TNNI3* | NM_000363.4 | c.434G>A | p.Arg145Gln | RCV000208273.1 / RCV000159223.4 | LP / P | CM971497 | HCM | DM | 0.00002490 | 0.000909 | rs397516349 |
| YMC-110 | *MYH6* | NM_002471.3 | c.679dupG | p.Ala227Glyfs*24 | (-) | (-) | (-) | (-) | (-) | 0.00000000 | 0.00000000 | Novel |
| YMC-114 | *MYBPC3* | NM_000256.3 | c.1000G>A | p.Glu334Lys | RCV000168764.2 | LP | CM073211 | HCM | DM | 0.00033786 | 0.006364 | rs573916965 |
|  | *MYH7* | NM_000257.3 | c.1324C>T | p.Arg442Cys | RCV000162335.1 / RCV000464365.1 | LP / P | CM066924 | HCM | DM | 0.00000824 | 0.00000000 | rs148808089 |
| YMC-117 | *TNNI3* | NM_000363.4 | c.434G>A | p.Arg145Gln | RCV000208273.1 / RCV000159223.4 | LP / P | CM971497 | HCM | DM | 0.00002490 | 0.000909 | rs397516349 |
| YMC-121 | *MYBPC3* | NM_000256.3 | c.1000G>A | p.Glu334Lys | RCV000168764.2 | LP | CM073211 | HCM | DM | 0.00033786 | 0.006364 | rs573916965 |
| YMC-123 | *TNNI3* | NM_000363.4 | c.434G>A | p.Arg145Gln | RCV000208273.1 / RCV000159223.4 | LP / P | CM971497 | HCM | DM | 0.00002490 | 0.000909 | rs397516349 |
| YMC-126 | *MYBPC3* | NM_000256.3 | c.1000G>A | p.Glu334Lys | RCV000168764.2 | LP | CM073211 | HCM | DM | 0.00033786 | 0.006364 | rs573916965 |
| YMC-127 | *MYBPC3* | NM_000256.3 | c.1000G>A | p.Glu334Lys | RCV000168764.2 | LP | CM073211 | HCM | DM | 0.00033786 | 0.006364 | rs573916965 |
| YMC-133 | *MYBPC3* | NM_000256.3 | c.2833_2834del | p.Arg945Glyfs | RCV000035530.2 | P | CD022562 | HCM | DM | 0.00000000 | 0.00000000 | rs397515987 |
| YMC-134 | *MYBPC3* | NM_000256.3 | c.2067+1G>A | (-) | (-) | (-) | CS063340 | HCM | DM | 0.00000000 | 0.00160800 | (-) |
| YMC-136 | *MYBPC3* | NM_000256.3 | c.2459G>A | p.Arg820Gln | RCV000158159.3/RCV000009148.6 | LP | CM034547 | HCM | DM | 0.00001657 | 0.00000000 | rs2856655 |
| YMC-137 | *TNNI3* | NM_000363.4 | c.433C>G | p.Arg145Gly | RCV000441050.1 / RCV000013231.25 | P | CM971498 | HCM | DM | 0.00000000 | 0.00000000 | rs104894724 |
| YMC-141 | *MYL3* | NM_000258.2 | c.170C>G | p.Ala57Gly | RCV000024471.3 / RCV000229595.2 | P | CM014210 | HCM | DM | 0.00000000 | 0.00160800 | rs139794067 |
| YMC-144 | *MYBPC3* | NM_000256.3 | c.1090+1G>A | (-) | RCV000382204.1/RCV000211795.1 | LP / P | CS068101 | HCM | DM | 0.00000000 | 0.00000000 | rs727504269 |
| YMC-148 | *MYH6* | NM_002471.3 | c.2384G>A | p.Arg795Gln | RCV000037469.3/RCV000171836.1/RCV000015210.21 | VUS / LP / P | CM020158 | HCM | DM | 0.00003298 | 0.000804 | rs267606907 |
| YMC-150 | TNNI3 | NM_000363.4 | c.434G>A | p.Arg145Gln | RCV000159223.3\|RCV000200141.2\|RCV000208273.1 | LP / P | CM971497 | HCM | DM | 0.0000249 | 0.000909 | rs397516349 |
| YMC-151 | MYBPC3 | NM_000256.3 | c.2067+1G>A | (-) | RCV000521997.1 | P | CS063340 | HCM | DM | 0.00000000 | 0.00160772 | (-) |
| YMC-154 | MYH7 | NM_000257.3 | c.1988G>A | p.Arg663His | RCV000035758.9 | P | CM993620 | HCM | DM | 0.00000000 | 0.00000000 | rs371898076 |
| YMC-159 | MYH7 | NM_000257.3 | c.1426C>T | p.Leu476Phe | (-) | (-) | (-) | (-) | (-) | 0.00000000 | 0.00000000 | Novel |
| YMC-164 | MYBPC3 | NM_000256.3 | c.2458C>T | p.Arg820Trp | RCV000176522.1 | LP | CM103890 | Cardiomyopathy | DM | 0.00000829 | 0.00000000 | rs775404728 |
| YMC-174 | MYBPC3 | NM_000256.3 | c.1000G>A | p.Glu334Lys | RCV000168764.2 | LP | CM073211 | HCM | DM | 0.00033786 | 0.006364 | rs573916965 |
| YMC-175 | MYH7 | NM_000257.3 | c.746G>A | p.Arg249Gln | RCV000158761.2/RCV000229956.1 | P | CM910268 | HCM | DM | 0.00000000 | 0.00000000 | rs3218713 |
| YMC-177 | MYH7 | NM_000257.3 | c.4123T>C | p.Tyr1375His | RCV000158634.1 | LP | CM167475 | HCM | DM | 0.00000000 | 0.00000000 | rs730880790 |
| YMC-180 | TNNI3 | NM_000363.4 | c.434G>A | p.Arg145Gln | RCV000200141.2/RCV000159223.3 | P | CM971497 | HCM | DM | 0.0000249 | 0.000909 | rs397516349 |
| YMC-189 | MYBPC3 | NM_000256.3 | c.3313_3314insGG | p.Ala1105Glyfs*85 | (-) | (-) | (-) | (-) | (-) | 0.00000000 | 0.00000000 | Novel |
| YMC-192 | MYBPC3 | NM_000256.3 | c.3034C>T | p.Gln1012* | RCV000158209.2 | P | CM034550 | HCM | DM | 0.00000000 | 0.00000000 | Novel |
| YMC-198 | MYH7 | NM_000257.3 | c.4066G>A | p.Glu1356Lys | RCV000168900.1 | LP | CM042422 | HCM | DM | 0.00000000 | 0.00000000 | rs727503246 |
| YMC-205 | MYBPC3 | NM_000256.3 | c.2833_2834delCG | p.Arg945Glyfs*105 | RCV000035530.2/RCV000158380.2 | P | CD022562 | HCM | DM | 0.00000000 | 0.00000000 | rs397515987 |
| YMC-206 | MYBPC3 | NM_000256.3 | c.2459G>A | p.Arg820Gln | RCV000158159.3/RCV000009148.6 | LP | CM034547 | HCM | DM | 0.0000166 | 0.00000000 | rs2856655 |
| YMC-207 | MYH7 | NM_000257.3 | c.1615A>G | p.Met539Val | RCV000158890.1/RCV000205072.1 | LP/P | CM1516537 | HCM | DM | 0.00000000 | 0.00000000 | rs730880930 |
| YMC-214 | MYH7 | NM_000257.3 | c.4130C>T | p.Thr1377Met | RCV000552931.1/RCV000518840.1/RCV000208315.2/RCV000617360.1 | VUS/LP | CM034558 | HCM | DM | 0.00000000 | 0.00000000 | rs397516201 |

Abbreviations: ACMG, American College of Medical Genetics and Genomics; dbSNP, database of single nucleotide polymorphism; D, D or D; DCM, dilated cardiomyopathy; DM, disease-causing mutation; ExAC, Exome Aggregation Consortium; HCM, hypertrophic cardiomyopathy; Het, Heterozygous; Hom, Homozygous; HGMD, human gene mutation database; KRGDB, Korean Reference Genome Database; LP, Likely pathogenic; MAF, minor allele frequency; NM number, National Center for Biotechnology Information (NCBI) reference sequence;OMGL/LMM, the Oxford Molecular Genetics Laboratory and the Laboratory of Molecular Medicine; P, pathogenic; T, Tolerated; VUS, variant of uncertain significance. ^a^Align GVGD (http://agvgd.iarc.fr/) was used as a nucleotide-conservation prediction algorithm. ^b^ In silico tools including SIFT (http://sift.jcvi.org) and MutationTaster (http://www.mutationtaster.org) were used to assess the predicted impact of missense change

**Supplementary Table S3.** Likely pathogenic or pathogenic variants in the 6 non-sarcomere genes and the 44 mitochondria-related nuclear genes

| Case | GENE | Reference sequence | DNA Change | Amino Acid Change | Align GVGD^a^ | SIFT^b^ | Mutation Taster^b^ | Clinvar accession | Clinvar phenotype | HGMD accession | HGMD phenotype | HGMD variant class | MAF (ExAC) | MAF (KRGDB) | dbSNP |
| --- | --- | --- | --- | --- | --- | --- | --- | --- | --- | --- | --- | --- | --- | --- | --- |
| YMC-12 | *AARS2* | NM_020745.3 | c.452T>C | p.Met151Thr | Class C0 (GV: 268.85 - GD: 38.84) | Tolerated (score: 0.07) | Disease causing (p-value: 1) | (-) | (-) | CM173156 | Ovarioleukodystrophy | DM | (-) | (-) | (-) |
|  | *ACADVL* | NM_000018.3 | c.865G>A | p.Gly289Arg | Class C0 (GV: 97.85 - GD: 42.00) | Deleterious (score: 0.01) | Disease causing (p-value: 1) | RCV000408960.1 | Very long chain acyl-CoA dehydrogenase deficiency | CM034380 | Very long chain acyl-CoA dehydrogenase deficiency | DM | 0.00009128 | 0.00045500 | rs200788251 |
| YMC-14 | *PCCB* | NM_000532.4 | c.1304A>G | p.Tyr435Cys | Class C55 (GV: 21.61 - GD: 191.71) | Deleterious (score: 0) | Disease causing (p-value: 1) | RCV000012798.24 | Propionic acidaemia | CM022051 | Propionic acidaemia | DM | 0.00004952 | 0.00318200 | rs121964961 |
| YMC-21 | *PCCB* | NM_000532.4 | c.1304A>G | p.Tyr435Cys | Class C55 (GV: 21.61 - GD: 191.71) | Deleterious (score: 0) | Disease causing (p-value: 1) | RCV000012798.24 | Propionic acidaemia | CM022051 | Propionic acidaemia | DM | 0.00004952 | 0.00318200 | rs121964961 |
| YMC-26 | *AGK* | NM_018238.3 | c.73G>T | p.Gly25* | (-) | (-) | (-) | (-) | (-) | (-) | (-) | (-) | (-) | (-) | (-) |
| YMC-38 | *GAA* | NM_001079803.2 | c.2171C>A | p.Ala724Asp | Class C0 (GV: 235.10 - GD: 79.30) | Deleterious (score: 0.04) | Disease causing (p-value: 1 | (-) | (-) | CM124040 | Glycogen storage disease 2 | DM | (-) | (-) | (-) |
| YMC-40 | *LRPPRC* | NM_133259.3 | c.4078G>A | p.Ala1360Thr | Class C0 (GV: 105.57 - GD: 26.10) | Deleterious (score: 0.04 | Disease causing (p-value: 1) | (-) | (-) | CM1715653 | Leigh syndrome | DM | 0.00078820 | 0.00884200 | rs147302249 |
| YMC-46 | *GTPBP3* | NM_032620.3 | c.8G>T | p.Arg3Leu | Class C0 (GV: 353.86 - GD: 0.00) | Tolerated (score: 0.08) | Disease causing (p-value: 0.74) | (-) | (-) | CM1413836 | Leigh syndrome | (-) | (-) | 0.00136400 | (-) |
| YMC-56 | *LRPPRC* | NM_133259.3 | c.3430C>T | p.Arg1144Cys | Class C0 (GV: 260.17 - GD: 0.00) | Tolerated (score: 0.15) | Disease causing (p-value: 1) | RCV000376700 | Leigh Syndrome | CM1715652 | Leigh syndrome | DM | 0.00014220 | (-) | rs760016065 |
| YMC-75 | *PCCB* | NM_000532.4 | c.1304A>G | p.Tyr435Cys | Class C55 (GV: 21.61 - GD: 191.71) | Deleterious (score: 0) | Disease causing (p-value: 1) | RCV000012798.24 | Propionic acidaemia | CM022051 | Propionic acidaemia | DM | 0.00004952 | 0.00318200 | rs121964961 |
| YMC-84 | *PCCB* | NM_000532.4 | c.1304A>G | p.Tyr435Cys | Class C55 (GV: 21.61 - GD: 191.71) | Deleterious (score: 0) | Disease causing (p-value: 1) | RCV000012798.24 | Propionic acidaemia | CM022051 | Propionic acidaemia | DM | 0.00004952 | 0.00318200 | rs121964961 |
| YMC-119 | *LRPPRC* | NM_133259.3 | c.4078G>A | p.Ala1360Thr | Class C0 (GV: 105.57 - GD: 26.10) | Deleterious (score: 0.04 | Disease causing (p-value: 1) | (-) | (-) | CM1715653 | Leigh syndrome | DM | 0.00078820 | 0.00884200 | rs147302249 |
| YMC-138 | *ACAD9* | NM_014049.4 | c.1552C>T | p.Arg518Cys | Class C0 (GV: 241.31 - GD: 79.30 | Deleterious (score: 0) | Disease causing (p-value: 1) | (-) | (-) | CM153909 | Acyl-Coenzyme dehydrogenase 9 deficiency | DM | 0.00010720 | (-) | rs150283105 |
| YMC-149 | *LRPPRC* | NM_133259.3 | c.4078G>A | p.Ala1360Thr | Class C0 (GV: 105.57 - GD: 26.10) | Deleterious (score: 0.04 | Disease causing (p-value: 1) | (-) | (-) | CM1715653 | Leigh syndrome | DM | 0.00078820 | 0.00884200 | rs147302249 |
| YMC-193 | *AARS2* | NM_020745.3 | c.609delT | p.Phe203Leufs*36 | (-) | (-) | (-) | (-) | (-) | (-) | (-) | (-) | 0.00000830 | (-) | (-) |
| YMC-194 | *PCCB* | NM_000532.4 | c.1304A>G | p.Tyr435Cys | Class C55 (GV: 21.61 - GD: 191.71) | Deleterious (score: 0) | Disease causing (p-value: 1) | RCV000012798.24 | Propionic acidaemia | CM022051 | Propionic acidaemia | DM | 0.00004952 | 0.00318200 | rs121964961 |
| YMC-196 | *PCCB* | NM_000532.4 | c.1304A>G | p.Tyr435Cys | Class C55 (GV: 21.61 - GD: 191.71) | Deleterious (score: 0) | Disease causing (p-value: 1) | RCV000012798.24 | Propionic acidaemia | CM022051 | Propionic acidaemia | DM | 0.00004952 | 0.00318200 | rs121964961 |

Abbreviations: ACMG, American College of Medical Genetics and Genomics; DCM, dilated cardiomyopathy; DM, disease-causing mutation; ExAC, Exome Aggregation Consortium; HCM, hypertrophic cardiomyopathy; HGMD, human gene mutation database; KRGDB, Korean Reference Genome Database; MAF, minor allele frequency; NM number, National Center for Biotechnology Information (NCBI) reference sequence; dbSNP, database of single nucleotide polymorphism; VUS, variant of uncertain significance. (Reprinted with permission from Science Direct.[52])

^a^Align GVGD (http://agvgd.iarc.fr/) was used as a nucleotide-conservation prediction algorithm.

^b^ In silico tools including SIFT (http://sift.jcvi.org) and MutationTaster (http://www.mutationtaster.org) were used to assess the predicted impact of missense change

**Supplementary Table S4.** Non-haplogroup-associated variants with a GenBank frequency < 0.1%

| Case | Gene |  | rCRS position | Homo  plasmy | Hetero  plasmy | Patients haplogroup | Variant haplogroup | Frequency GenBank^a^ | PolyPhen2^b^ | SIFT^b^ | PROVEAN^b^ | dbSNP | Associated_disease | Mitomap status | Conservation |
| --- | --- | --- | --- | --- | --- | --- | --- | --- | --- | --- | --- | --- | --- | --- | --- |
| YMC-3 | *MT-TT* |  | m.15926C>T | + |  | R0+16189 | (-) | 0.0002 | . | . | . | . | . | . | 95.56% |
| YMC-8 | *MT-RNR1* |  | m.1118A>T | + |  | c | (-) | 0.0000 | . | . | . | . | . | . | 53.33% |
| **YMC-10** | ***MT-ATP8*** |  | **m.8381A>G** | **+** |  | **M7b1a1b** | **(-)** | **0.0002** | **probably_damaging** | **neutral** | **deleterious** | **.** | **MIDD / LVNC cardiomyopathy.assoc,** | **Pathogenic** | **84.44%** |
| YMC-15 | *MT-CYB* |  | m.15203A>G | + |  | R0+16189 | (-) | 0.0002 | benign | neutral | neutral | rs193302988 | . | . | 86.67% |
| YMC-16 | *MT-ATP8* |  | m.8547T>C | + |  | M7c1a3 | (-) | 0.0003 | probably_damaging | neutral | neutral | . | . | . | 13.33% |
| YMC-18 | *MT-TR* |  | m.10455A>G | + |  | F1a3a1a | (-) | 0.0003 | . | . | . | . | . | . | 88.89% |
| YMC-24 | *MT-ND2* |  | m.5086C>T | + |  | N9a3 | (-) | 0.0001 | benign | neutral | neutral | . | . | . | 33.33% |
| YMC-24 | *MT-TH* |  | m.12190A>G | + |  | N9a3 | (-) | 0.0003 | . | . | . | . | . | . | 62.22% |
| YMC-25 | *MT-RNR1* |  | m.792C>T | + |  | G2b2 | (-) | 0.0001 | . | . | . | . | Increased risk of nonsyndromic deafness | Reported^c^ | 95.56% |
| YMC-25 | *MT-TA* |  | m.5641T>C | + |  | G2b2 | (-) | 0.0001 | . | . | . | . | . | . | 97.78% |
| YMC-27 | *MT-RNR2* |  | m.1709G>C | + |  | F1b1a1a2 | (-) | 0.0000 | . | . | . | . | . | . | 2.22% |
| YMC-34 | *MT-TF* |  | m.603A>G | + |  | D4j | (-) | 0.0000 | . | . | . | . | . | . | 91.11% |
| YMC-35 | *MT-ND4L* |  | m.10680G>A | + |  | D4a1b | (-) | 0.0004 | benign | neutral | deleterious | rs386829109 | LHON | Reported^c^ | 93.33% |
| **YMC-36** | ***MT-TL1*** |  | **m.3243A>G** |  | **+** | **C1a** | **(-)** | **0.0002** | **.** | **.** | **.** | **.** | **MELAS /MIDD / Cardiac+multi.organ dysfunction** | **Pathogenic** | **97.78%** |
| YMC-36 | *MT-ATP6* |  | m.9099C>A | + |  | C1a | (-) | 0.0000 | probably_damaging | neutral | neutral | . | . | . | 75.56% |
| YMC-36 | *MT-CYB* |  | m.15773G>A | + |  | C1a | V2b1 | 0.0010 | probably_damaging | neutral | neutral | . | LHON | Reported^c^ | 100.00% |
| YMC-37 | *MT-CYB* |  | m.15381C>T | + |  | R0+16189 | (-) | 0.0003 | benign | neutral | neutral | rs199721378 | . | . | 15.56% |
| YMC-40 | *MT-ND5* |  | m.13942A>G | + |  | R0+16189 | B4d | 0.0010 | benign | neutral | neutral | rs200657506 | . | . | 11.11% |
| YMC-40 | *MT-ND5* |  | m.14003C>T | + |  | R0+16189 | U5a1i1 | 0.0004 | benign | neutral | neutral | rs386829198 | . | . | 28.89% |
| YMC-42 | *MT-ND2* |  | m.5086C>T | + |  | N9a3 | (-) | 0.0001 | benign | neutral | neutral | . | . | . | 33.33% |
| YMC-42 | *MT-TH* |  | m.12190A>G | + |  | N9a3 | (-) | 0.0003 | . | . | . | . | . | . | 62.22% |
| YMC-48 | *MT-CYB* |  | m.15113A>G | + |  | D4j3 | (-) | 0.0003 | probably_damaging | neutral | neutral | . | . | . | 33.33% |
| YMC-50 | *MT-TV* |  | m.1654TA>T | + |  | M7b1a1a1b | (-) | 0.0000 | . | . | . | . | . | . | 15.56% |
| YMC-54 | *MT-CYB* |  | m.15779T>C |  | + | D5b1b1 | (-) | 0.0002 | benign | neutral | neutral | . | . | . | 22.22% |
| YMC-55 | *MT-ND4* |  | m.11729T>C |  | + | Z4a | (-) | 0.0000 | probably_damaging | neutral | deleterious | . | . | . | 100.00% |
| YMC-55 | *ATT* |  | m.15939CT>C | + |  | Z4a | (-) | 0.0000 | . | . | . | . | . | . | 22.22% |
| YMC-58 | *MT-TA* |  | m.5641T>C | + |  | G2b2 | (-) | 0.0001 | . | . | . | . | . | . | 97.78% |
| **YMC-58** | ***MT-ND4*** |  | **m.11984T>C** | **+** |  | **G2b2** | **L1c3c, M35c, U5a2b2** | **0.0009** | **benign** | **neutral** | **deleterious** | **rs200911567** | **Leigh Disease** | **Pathogenic** | **88.89%** |
| YMC-60 | *MT-CYB* |  | m.15434C>T | + |  | B4b1c1 | (-) | 0.0002 | probably_damaging | neutral | neutral | . | . | . | 93.33% |
| YMC-62 | *MT-ND4* |  | m.11372G>A | + |  | F2a | (-) | 0.0000 | benign | neutral | neutral | . | . | . | 86.67% |
| YMC-63 | *MT-CO3* |  | m.9612G>T | + |  | G2a1d1 | (-) | 0.0000 | probably_damaging | neutral | neutral | . | . | . | 86.67% |
| YMC-64 | *MT-RNR1* |  | m.942A>G | + |  | D5a2a1a | (-) | 0.0007 | . | . | . | . | . | . | 35.56% |
| YMC-64 | *MT-ND5* |  | m.12398C>T | + |  | D5a2a1a | M41 | 0.0005 | unknown | neutral | neutral | . | . | . | 20.00% |
| YMC-69 | *MT-CO2* |  | m.7962T>C | + |  | B4f1 | (-) | 0.0001 | probably_damaging | neutral | neutral | rs373420717 | . | . | 20.00% |
| YMC-74 | *MT-TR* |  | m.10455A>G | + |  | F1a3a1a | (-) | 0.0003 | . | . | . | . | . | . | 88.89% |
| YMC-80 | *MT-ATP6* |  | m.9081C>T | + |  | M9a1a1a | (-) | 0.0002 | . | . | . | . | . | . | 26.67% |
| YMC-81 | *MT-ND3* |  | m.10192C>A | + |  | R0+16189 | (-) | 0.0001 | benign | neutral | deleterious | . | . | . | 17.78% |
| YMC-81 | *MT-CYB* |  | m.15381C>T | + |  | R0+16189 | (-) | 0.0003 | benign | neutral | neutral | rs199721378 | . | . | 15.56% |
| YMC-86 | *MT-ATP6* |  | m.9182G>A | + |  | A+152+16362 | (-) | 0.0008 | probably_damaging | neutral | neutral | . | . | . | 95.56% |
| YMC-90 | *MT-RNR2* |  | m.2881C>T |  | + | F1a1 | (-) | 0.0000 | . | . | . | . | . | . | 20.00% |
| YMC-90 | *MT-TG* |  | m.10043C>T | + |  | F1a1 | (-) | 0.0001 | . | . | . | . | . | . | 26.67% |
| YMC-91 | *MT-CYB* |  | m.15363A>G | + |  | F1a1 | (-) | 0.0001 | probably_damaging | neutral | deleterious | . | . | . | 93.33% |
| YMC-94 | *MT-RNR2* |  | m.2399A>G | + |  | F2 | (-) | 0.0000 | . | . | . | . | . | . | 80.00% |
| YMC-94 | *MT-ATP6* |  | m.8654T>C | + |  | F2 | (-) | 0.0004 | benign | neutral | neutral | rs200811540 | . | . | 48.89% |
| YMC-99 | *MT-CO1* |  | m.7278T>C | + |  | M8a2c | (-) | 0.0009 | benign | neutral | neutral | . | . | . | 86.67% |
| YMC-100 | *MT-RNR2* |  | m.2226T>C | + |  | C5a1 | (-) | 0.0004 | . | . | . | . | . | . | 42.22% |
| **YMC-102** | ***MT-CO2*** |  | **m.8078G>A** | **+** |  | **C4a1** | **J2a2b3** | **0.0006** | **benign** | **neutral** | **neutral** | **.** | **DEAF** | **Pathogenic** | **84.44%** |
| YMC-104 | *MT-RNR2* |  | m.2226T>C | + |  | C5a1 | (-) | 0.0004 | . | . | . | . | . | . | 42.22% |
| YMC-108 | *MT-RNR2* |  | m.2124A>G | + |  | D4a1 | (-) | 0.0002 | . | . | . | . | . | . | 64.44% |
| YMC-109 | *MT-RNR2* |  | m.2244T>C | + |  | B5a2a1+16129 | (-) | 0.0003 | . | . | . | . | . | . | 20.00% |
| YMC-111 | *MT-CO3* |  | m.9337T>C | + |  | D4g1c | (-) | 0.0002 | benign | neutral | neutral | . | . | . | 15.56% |
| **YMC-113** | ***MT-TI*** |  | **m.4317A>G** | **+** |  | **D5c2** | **(-)** | **0.0005** | **.** | **.** | **.** | **.** | **Cardiomyopathy** | **Reported^c^** | **71.11%** |
| YMC-113 | *MT-TW* |  | m.5554C>T | + |  | D5c2 | (-) | 0.0001 | . | . | . | . | . | . | 15.56% |
| YMC-117 | *MT-ATP6* |  | m.8945T>C | + |  | D4e2a | (-) | 0.0003 | probably_damaging | deleterious | deleterious | . | . | . | 86.67% |
| YMC-118 | *MT-CO1* |  | m.6481T>C | + |  | G2a1+16189+16194 | (-) | 0.0001 | benign | deleterious | deleterious | rs28721398 | . | . | 88.89% |
| YMC-119 | *MT-TT* |  | m.15926C>T | + |  | R0+16189 | (-) | 0.0002 | . | . | . | . | . | . | 95.56% |
| YMC-121 | *MT-ND3* |  | m.10306A>G | + |  | F1b1a1 | (-) | 0.0000 | benign | neutral | neutral | . | . | . | 73.33% |
| YMC-122 | *MT-ND1* |  | m.4231A>G | + |  | M9a | H13a1b | 0.0004 | benign | neutral | neutral | . | . | . | 80.00% |
| YMC-124 | *MT-CYB* |  | m.14954A>G | + |  | D4j | (-) | 0.0002 | probably_damaging | neutral | neutral | . | . | . | 22.22% |
| YMC-129 | *MT-RNR2* |  | m.1699C>T |  | + | D4a1 | (-) | 0.0000 | . | . | . | . | . | . | 20.00% |
| YMC-130 | *MT-TS1* |  | m.7465AC>A | + |  | D4a1 | (-) | 0.0000 | . | . | . | . | . | . | 100.00% |
| YMC-131 | *MT-RNR2* |  | m.2857T>C | + |  | D4j | (-) | 0.0009 | . | . | . | . | . | . | 44.44% |
| YMC-131 | *MT-CYB* |  | m.14954A>G | + |  | D4j | (-) | 0.0002 | probably_damaging | neutral | neutral | . | . | . | 22.22% |
| YMC-133 | *MT-RNR1* |  | m.1118A>T | + |  | C | (-) | 0.0000 | . | . | . | . | . | . | 53.33% |
| YMC-134 | *MT-ATP6* |  | m.9187T>C | + |  | D4b1b | (-) | 0.0000 | probably_damaging | deleterious | deleterious | . | . | . | 100.00% |
| YMC-136 | *MT-CO2* |  | m.7980A>G | + |  | R11b | (-) | 0.0001 | probably_damaging | neutral | neutral | . | . | . | 28.89% |
| YMC-139 | *MT-ATP6* |  | m.9182G>A |  | + | D5b1b2 | (-) | 0.0008 | probably_damaging | neutral | neutral | . | . | . | 95.56% |
| YMC-142 | *MT-ATP6* |  | m.8711A>G | + |  | N9a1 | (-) | 0.0003 | benign | neutral | deleterious | . | . | . | 80.00% |
| YMC-147 | *MT-RNR2* |  | m.2978T>C |  | + | F1b1a2 | (-) | 0.0000 | . | . | . | . | . | . | 95.56% |
| YMC-151 | MT-RNR2 |  | m.3083T>C | + | . | M10a1a1a | (-) | 0.0007 | . | . | . | . | . | . | 55.56% |
| YMC-151 | MT-ND6 |  | m.14337C>T | + | . | M10a1a1a | (-) | 0.0001 | probably_damaging | neutral | neutral | - | . | . | 64.44% |
| YMC-154 | MT-RNR2 |  | m.2216A>G | + | . | B5b1 | (-) | 0.0000 | . | . | . | . | . | . | 73.33% |
| YMC0156 | MT-ATP8 |  | m.8516T>C | + | . | N9a9 | (-) | 0.0002 | probably_damaging | neutral | deleterious | - | . | . | 97.78% |
| YMC-157 | MT-ATP6 |  | m.8597T>C | + | . | D4g1a | (-) | 0.0004 | probably_damaging | neutral | deleterious | - | . | . | 71.11% |
| YMC-159 | MT-RNR2 |  | m.1699C>T | + | . | D4a6 | (-) | 0.0000 | . | . | . | . | . | . | 20.00% |
| YMC-161 | MT-RNR2 |  | m.3169C>T | + | . | B4c1c | (-) | 0.0002 | . | . | . | . | . | . | 31.11% |
| YMC-164 | MT-RNR2 |  | m.2857T>C | + | . | D4j | (-) | 0.0009 | . | . | . | . | . | . | 44.44% |
| YMC-164 | MT-CYB |  | m.14954A>G | + | . | D4j | (-) | 0.0002 | probably_damaging | neutral | neutral | - | . | . | 22.22% |
| YMC-166 | MT-CO1 |  | m.7245A>G | + | . | D4g1 | (-) | 0.0007 | probably_damaging | neutral | neutral | rs386829003 | . | . | 97.78% |
| YMC-167 | MT-HV2 |  | m.227A>T | + | . | M7c1a2a1 | (-) | 0.0001 | . | . | . | . | . | . |  |
| YMC-167 | MT-RNR2 |  | m.2861A>G |  | + | M7c1a2a1 | (-) | 0.0000 | . | . | . | . | . | . | 82.22% |
| YMC-167 | MT-CYB |  | m.15038A>G | + | . | M7c1a2a1 | (-) | 0.0006 | possibly_damaging | neutral | neutral | rs202045169 | . | . | 33.33% |
| YMC-174 | MT-HV2 |  | m.333T>C | + | . | B4b1b | (-) | 0.0003 | . | . | . | . | . | . |  |
| YMC-174 | MT-RNR2 |  | m.1811A>T |  | + | B4b1b | (-) | 0.0000 | . | . | . | . | . | . | 22.22% |
| YMC-175 | MT-RNR1 |  | m.1235T>C | + | . | A5a | (-) | 0.0001 | . | . | . | . | . | . | 77.78% |
| YMC-181 | MT-TFX |  | m.252T>C | + | . | D5b1b | (-) | 0.0008 | . | . | . | . | . | . |  |
| YMC-183 | MT-ATP6 |  | m.9025G>A | + | . | D5a2a1b | (-) | 0.0006 | probably_damaging | deleterious | deleterious | rs28681063 | . | . | 100.00% |
| YMC-184 | MT-HV2 |  | m.65T>C | + | . | B4c1b2a | (-) | 0.0001 | . | . | . | . | . | . |  |
| YMC-184 | MT-CO2 |  | m.7773C>T | + | . | B4c1b2a | (-) | 0.0001 | benign | neutral | neutral | - | . | . | 86.67% |
| YMC-184 | MT-ATP6 |  | m.9139G>A |  | + | B4c1b2a | (-) | 0.0008 | probably_damaging | neutral | deleterious | - | LHON | Reported^c^ | 95.56% |
| YMC-186 | MT-HV2 |  | m.290A>G | + | . | D4a | (-) | 0.0000 | . | . | . | . | . | . |  |
| YMC-188 | MT-ATP6 |  | m.9179T>C | + | . | A5a3a | (-) | 0.0000 | probably_damaging | neutral | deleterious | - | . | . | 84.44% |
| YMC-198 | MT-CO1 |  | m.6747T>C |  | + | A1 | (-) | 0.0000 | probably_damaging | neutral | neutral | - | . | . | 93.33% |
| YMC-198 | MT-ATP6 |  | m.8908T>C | + | . | A1 | (-) | 0.0002 | benign | neutral | neutral | - | . | . | 66.67% |
| YMC-205 | MT-RNR2 |  | m.1676A>G | + | . | B5b2a1 | (-) | 0.0003 | . | . | . | . | . | . | 55.56% |
| YMC-205 | MT-CO1 |  | m.7356G>A | + | . | B5b2a1 | (-) | 0.0008 | benign | neutral | neutral | - | . | . | 64.44% |
| YMC-208 | MT-RNR2 |  | m.3083T>C | + | . | D4b1b | (-) | 0.0006 | . | . | . | . | . | . | 55.56% |
| YMC-208 | MT-NC3 |  | m.5584A>G | + | . | D4b1b | (-) | 0.0006 | . | . | . | . | . | . | . |
| YMC-208 | MT-ATP6 |  | m.9187T>C | + | . | D4b1b | (-) | 0.0000 | probably_damaging | deleterious | deleterious | - | . | . | 100.00% |
| YMC-208 | MT-ND5 |  | m.13153A>G | + | . | D4b1b | (-) | 0.0004 | benign | neutral | neutral | - | . | . | 51.11% |
| YMC-214 | MT-HV2 |  | m.209T>C | + | . | N9a5 | (-) | 0.0002 | . | . | . | . | . | . | . |
| YMC-215 | MT-CO1 |  | m.6735A>G | + | . | F2d | (-) | 0.0000 | benign | neutral | neutral | - | . | . | 82.22% |
| YMC-216 | MT-HV2 |  | m.352A>C |  | + | N9a2 | (-) | 0.0000 | . | . | . | . | . | . | . |
| YMC-216 | MT-TN |  | m.5715A>G | + | . | N9a2 | (-) | 0.0002 | . | . | . | . | . | . | 82.22% |

Abbreviations: DEAF, Maternally inherited deafness or aminoglycoside-induced deafness; dbSNP, database of single nucleotide polymorphism; Homoplasmy,pure mutant mtDNA; Heteroplasmy, mixture of mutant and normal mtDNA; LHON, Leber Hereditary Optic Neuropathy; LVNC, Left ventricular noncompaction; MELAS, Mitochondrial Encephalomyopathy, Lactic Acidosis, and Stroke-like episodes; MIDD, Maternally Inherited Diabetes and Deafness. (Reprinted with permission from Science Direct.[52])

^a^The GB frequency data is derived from 45,494 full length (FL) human mitochondrial sequences (>15.4 kbp) from GenBank.

^b^ In silico tools including SIFT (http://sift.jcvi.org), PolyPhen2 (http://genetics.bwh.harvard.edu/pph2/) and PROVEAN (http://provean.jcvi.org/index.php) were used to assess the predicted impact of missense change

^c^Reported" status indicates that one or more publications have considered the mutation as possibly pathologic.

Rows in bold are pathogenic variants in MITOMAP database (https://www.mitomap.org/foswiki/bin/view/MITOMAP/WebHome) and putative pathogenic variants associated with cardiomyopathy.

**References**

[1] Wang HW, Jia X, Ji Y, Kong QP, Zhang Q, Yao YG, et al. Strikingly different penetrance of LHON in two Chinese families with primary mutation G11778A is independent of mtDNA haplogroup background and secondary mutation G13708A. Mutat Res. 2008;643:48-53.

[2] Andrews RM, Kubacka I, Chinnery PF, Lightowlers RN, Turnbull DM, Howell N. Reanalysis and revision of the Cambridge reference sequence for human mitochondrial DNA. Nat Genet. 1999;23:147.

[3] Li H, Homer N. A survey of sequence alignment algorithms for next-generation sequencing. Brief Bioinform. 2010;11:473-83.

[4] Brandon MC, Lott MT, Nguyen KC, Spolim S, Navathe SB, Baldi P, et al. MITOMAP: a human mitochondrial genome database--2004 update. Nucleic Acids Res. 2005;33:D611-3.

[5] Ingman M, Gyllensten U. mtDB: Human Mitochondrial Genome Database, a resource for population genetics and medical sciences. Nucleic Acids Res. 2006;34:D749-51.

[6] Chaitanya L, Ralf A, van Oven M, Kupiec T, Chang J, Lagace R, et al. Simultaneous Whole Mitochondrial Genome Sequencing with Short Overlapping Amplicons Suitable for Degraded DNA Using the Ion Torrent Personal Genome Machine. Hum Mutat. 2015;36:1236-47.

[7] Zaragoza MV, Brandon MC, Diegoli M, Arbustini E, Wallace DC. Mitochondrial cardiomyopathies: how to identify candidate pathogenic mutations by mitochondrial DNA sequencing, MITOMASTER and phylogeny. European journal of human genetics : EJHG. 2011;19:200-7.

[8] Amano Y, Kitamura M, Tachi M, Takeda M, Mizuno K, Kumita S. Delayed enhancement magnetic resonance imaging in hypertrophic cardiomyopathy with Basal septal hypertrophy and preserved ejection fraction: relationship with ventricular tachyarrhythmia. Journal of computer assisted tomography. 2014;38:67-71.

[9] Schulz-Menger J, Bluemke DA, Bremerich J, Flamm SD, Fogel MA, Friedrich MG, et al. Standardized image interpretation and post processing in cardiovascular magnetic resonance: Society for Cardiovascular Magnetic Resonance (SCMR) board of trustees task force on standardized post processing. J Cardiovasc Magn Reson. 2013;15:35.

[10] Walsh R, Buchan R, Wilk A, John S, Felkin LE, Thomson KL, et al. Defining the genetic architecture of hypertrophic cardiomyopathy: re-evaluating the role of non-sarcomeric genes. European heart journal. 2017;38:3461-8.

[11] Lee HC, Cherk SW, Chan SK, Wong S, Tong TW, Ho WS, et al. BAG3-related myofibrillar myopathy in a Chinese family. Clinical genetics. 2012;81:394-8.

[12] Landstrom AP, Ackerman MJ. Beyond the cardiac myofilament: hypertrophic cardiomyopathy- associated mutations in genes that encode calcium-handling proteins. Current molecular medicine. 2012;12:507-18.

[13] Li MX, Hwang PM. Structure and function of cardiac troponin C (TNNC1): Implications for heart failure, cardiomyopathies, and troponin modulating drugs. Gene. 2015;571:153-66.

[14] Gigli M, Begay RL, Morea G, Graw SL, Sinagra G, Taylor MR, et al. A Review of the Giant Protein Titin in Clinical Molecular Diagnostics of Cardiomyopathies. Frontiers in cardiovascular medicine. 2016;3:21.

[15] Yingchoncharoen T, Tang WW. Recent advances in hypertrophic cardiomyopathy. F1000prime reports. 2014;6:12.

[16] Vermeer AMC, Janssen A, Boorsma PC, Mannens M, Wilde AAM, Christiaans I. Transthyretin amyloidosis: a phenocopy of hypertrophic cardiomyopathy. Amyloid : the international journal of experimental and clinical investigation : the official journal of the International Society of Amyloidosis. 2017;24:87-91.

[17] Gotz A, Tyynismaa H, Euro L, Ellonen P, Hyotylainen T, Ojala T, et al. Exome sequencing identifies mitochondrial alanyl-tRNA synthetase mutations in infantile mitochondrial cardiomyopathy. American journal of human genetics. 2011;88:635-42.

[18] Haack TB, Danhauser K, Haberberger B, Hoser J, Strecker V, Boehm D, et al. Exome sequencing identifies ACAD9 mutations as a cause of complex I deficiency. Nature genetics. 2010;42:1131-4.

[19] Mathur A, Sims HF, Gopalakrishnan D, Gibson B, Rinaldo P, Vockley J, et al. Molecular heterogeneity in very-long-chain acyl-CoA dehydrogenase deficiency causing pediatric cardiomyopathy and sudden death. Circulation. 1999;99:1337-43.

[20] Mayr JA, Haack TB, Graf E, Zimmermann FA, Wieland T, Haberberger B, et al. Lack of the mitochondrial protein acylglycerol kinase causes Sengers syndrome. American journal of human genetics. 2012;90:314-20.

[21] Papadopoulou LC, Sue CM, Davidson MM, Tanji K, Nishino I, Sadlock JE, et al. Fatal infantile cardioencephalomyopathy with COX deficiency and mutations in SCO2, a COX assembly gene. Nature genetics. 1999;23:333-7.

[22] Baertling F, M AMvdB, Hertecant JL, Al-Shamsi A, L PvdH, Distelmaier F, et al. Mutations in COA6 cause cytochrome c oxidase deficiency and neonatal hypertrophic cardiomyopathy. Human mutation. 2015;36:34-8.

[23] Sondheimer N, Hewson S, Cameron JM, Somers GR, Broadbent JD, Ziosi M, et al. Novel recessive mutations in COQ4 cause severe infantile cardiomyopathy and encephalopathy associated with CoQ10 deficiency. Molecular genetics and metabolism reports. 2017;12:23-7.

[24] Antonicka H, Leary SC, Guercin GH, Agar JN, Horvath R, Kennaway NG, et al. Mutations in COX10 result in a defect in mitochondrial heme A biosynthesis and account for multiple, early-onset clinical phenotypes associated with isolated COX deficiency. Human molecular genetics. 2003;12:2693-702.

[25] Bourens M, Barrientos A. A CMC1-knockout reveals translation-independent control of human mitochondrial complex IV biogenesis. EMBO reports. 2017;18:477-94.

[26] Schiff M, Ogier de Baulny H, Lombes A. Neonatal cardiomyopathies and metabolic crises due to oxidative phosphorylation defects. Seminars in fetal & neonatal medicine. 2011;16:216-21.

[27] Abdulhag UN, Soiferman D, Schueler-Furman O, Miller C, Shaag A, Elpeleg O, et al. Mitochondrial complex IV deficiency, caused by mutated COX6B1, is associated with encephalomyopathy, hydrocephalus and cardiomyopathy. European journal of human genetics : EJHG. 2015;23:159-64.

[28] Isackson PJ, Bennett MJ, Lichter-Konecki U, Willis M, Nyhan WL, Sutton VR, et al. CPT2 gene mutations resulting in lethal neonatal or severe infantile carnitine palmitoyltransferase II deficiency. Molecular genetics and metabolism. 2008;94:422-7.

[29] Haack TB, Jackson CB, Murayama K, Kremer LS, Schaller A, Kotzaeridou U, et al. Deficiency of ECHS1 causes mitochondrial encephalopathy with cardiac involvement. Annals of clinical and translational neurology. 2015;2:492-509.

[30] Haack TB, Kopajtich R, Freisinger P, Wieland T, Rorbach J, Nicholls TJ, et al. ELAC2 mutations cause a mitochondrial RNA processing defect associated with hypertrophic cardiomyopathy. Am J Hum Genet. 2013;93:211-23.

[31] Fassone E, Duncan AJ, Taanman JW, Pagnamenta AT, Sadowski MI, Holand T, et al. FOXRED1, encoding an FAD-dependent oxidoreductase complex-I-specific molecular chaperone, is mutated in infantile-onset mitochondrial encephalopathy. Human molecular genetics. 2010;19:4837-47.

[32] Kopajtich R, Nicholls TJ, Rorbach J, Metodiev MD, Freisinger P, Mandel H, et al. Mutations in GTPBP3 cause a mitochondrial translation defect associated with hypertrophic cardiomyopathy, lactic acidosis, and encephalopathy. American journal of human genetics. 2014;95:708-20.

[33] Orii KE, Aoyama T, Wakui K, Fukushima Y, Miyajima H, Yamaguchi S, et al. Genomic and mutational analysis of the mitochondrial trifunctional protein beta-subunit (HADHB) gene in patients with trifunctional protein deficiency. Human molecular genetics. 1997;6:1215-24.

[34] Olahova M, Hardy SA, Hall J, Yarham JW, Haack TB, Wilson WC, et al. LRPPRC mutations cause early-onset multisystem mitochondrial disease outside of the French-Canadian population. Brain : a journal of neurology. 2015;138:3503-19.

[35] Galmiche L, Serre V, Beinat M, Assouline Z, Lebre AS, Chretien D, et al. Exome sequencing identifies MRPL3 mutation in mitochondrial cardiomyopathy. Human mutation. 2011;32:1225-31.

[36] Saada A, Shaag A, Arnon S, Dolfin T, Miller C, Fuchs-Telem D, et al. Antenatal mitochondrial disease caused by mitochondrial ribosomal protein (MRPS22) mutation. Journal of medical genetics. 2007;44:784-6.

[37] Ghezzi D, Baruffini E, Haack TB, Invernizzi F, Melchionda L, Dallabona C, et al. Mutations of the mitochondrial-tRNA modifier MTO1 cause hypertrophic cardiomyopathy and lactic acidosis. American journal of human genetics. 2012;90:1079-87.

[38] Hoefs SJ, van Spronsen FJ, Lenssen EW, Nijtmans LG, Rodenburg RJ, Smeitink JA, et al. NDUFA10 mutations cause complex I deficiency in a patient with Leigh disease. European journal of human genetics : EJHG. 2011;19:270-4.

[39] Berger I, Hershkovitz E, Shaag A, Edvardson S, Saada A, Elpeleg O. Mitochondrial complex I deficiency caused by a deleterious NDUFA11 mutation. Annals of neurology. 2008;63:405-8.

[40] Dunning CJ, McKenzie M, Sugiana C, Lazarou M, Silke J, Connelly A, et al. Human CIA30 is involved in the early assembly of mitochondrial complex I and mutations in its gene cause disease. The EMBO journal. 2007;26:3227-37.

[41] Loeffen J, Elpeleg O, Smeitink J, Smeets R, Stockler-Ipsiroglu S, Mandel H, et al. Mutations in the complex I NDUFS2 gene of patients with cardiomyopathy and encephalomyopathy. Annals of neurology. 2001;49:195-201.

[42] Budde SM, van den Heuvel LP, Janssen AJ, Smeets RJ, Buskens CA, DeMeirleir L, et al. Combined enzymatic complex I and III deficiency associated with mutations in the nuclear encoded NDUFS4 gene. Biochemical and biophysical research communications. 2000;275:63-8.

[43] Loeffen J, Smeitink J, Triepels R, Smeets R, Schuelke M, Sengers R, et al. The first nuclear-encoded complex I mutation in a patient with Leigh syndrome. American journal of human genetics. 1998;63:1598-608.

[44] Lee TM, Addonizio LJ, Barshop BA, Chung WK. Unusual presentation of propionic acidaemia as isolated cardiomyopathy. Journal of inherited metabolic disease. 2009;32 Suppl 1:S97-101.

[45] Alston CL, Ceccatelli Berti C, Blakely EL, Olahova M, He L, McMahon CJ, et al. A recessive homozygous p.Asp92Gly SDHD mutation causes prenatal cardiomyopathy and a severe mitochondrial complex II deficiency. Human genetics. 2015;134:869-79.

[46] Ohkuma A, Noguchi S, Sugie H, Malicdan MC, Fukuda T, Shimazu K, et al. Clinical and genetic analysis of lipid storage myopathies. Muscle & nerve. 2009;39:333-42.

[47] Iacobazzi V, Invernizzi F, Baratta S, Pons R, Chung W, Garavaglia B, et al. Molecular and functional analysis of SLC25A20 mutations causing carnitine-acylcarnitine translocase deficiency. Human mutation. 2004;24:312-20.

[48] Korver-Keularts IM, de Visser M, Bakker HD, Wanders RJ, Vansenne F, Scholte HR, et al. Two Novel Mutations in the SLC25A4 Gene in a Patient with Mitochondrial Myopathy. JIMD reports. 2015;22:39-45.

[49] Wedatilake Y, Brown RM, McFarland R, Yaplito-Lee J, Morris AA, Champion M, et al. SURF1 deficiency: a multi-centre natural history study. Orphanet journal of rare diseases. 2013;8:96.

[50] Powell CA, Kopajtich R, D'Souza AR, Rorbach J, Kremer LS, Husain RA, et al. TRMT5 Mutations Cause a Defect in Post-transcriptional Modification of Mitochondrial tRNA Associated with Multiple Respiratory-Chain Deficiencies. American journal of human genetics. 2015;97:319-28.

[51] Shahni R, Wedatilake Y, Cleary MA, Lindley KJ, Sibson KR, Rahman S. A distinct mitochondrial myopathy, lactic acidosis and sideroblastic anemia (MLASA) phenotype associates with YARS2 mutations. American journal of medical genetics Part A. 2013;161a:2334-8.

[52] Chung H, Kim Y, Cho SM, Lee HJ, Park CH, Kim JY, et al. Differential contributions of sarcomere and mitochondria-related multigene variants to the endophenotype of hypertrophic cardiomyopathy. Mitochondrion. 2020;53:48-56.
